# Silver-assisted reduction of nitroarenes by an Ag-embedded curcumin/melamine-functionalized magnetic nanocatalyst

**DOI:** 10.1038/s41598-023-32560-1

**Published:** 2023-03-30

**Authors:** Nima Khaleghi, Mohadeseh Forouzandeh-Malati, Fatemeh Ganjali, Zahra Rashvandi, Simindokht Zarei-Shokat, Reza Taheri-Ledari, Ali Maleki

**Affiliations:** Catalysts and Organic Synthesis Research Laboratory, Department of Chemistry, Iran University of Science and Technology, Tehran, 16846-13114 Iran

**Keywords:** Environmental sciences, Natural hazards, Chemistry, Materials science

## Abstract

In the current study, we introduce a hybrid magnetic nanocomposite comprised of curcumin (Cur), iron oxide magnetic nanoparticles (Fe_3_O_4_ MNPs), melamine linker (Mel), and silver nanoparticles (Ag NPs). Initially, a facile in situ route is administrated for preparing the Fe_3_O_4_@Cur/Mel-Ag effectual magnetic catalytic system. In addition, the advanced catalytic performance of the nanocomposite to reduce the nitrobenzene (NB) derivatives as hazardous chemical substances were assessed. Nevertheless, a high reaction yield of 98% has been achieved in short reaction times 10 min. Moreover, the Fe_3_O_4_@Cur/Mel-Ag magnetic nanocomposite was conveniently collected by an external magnet and recycled 5 times without a noticeable diminish in catalytic performance. Therefore, the prepared magnetic nanocomposite is a privileged substance for NB derivatives reduction since it achieved notable catalytic activity.

## Introduction

In line with environmental research, pollutant elimination from natural resources has become a significant challenge and global concern^1–3^. This concern has increased over the past decade in proportion to increased industrial activity and the release of waste into water resources^4,5^. Among various harmful species of water pollutants, nitrobenzene (NB), derived from industrial sources such as pharmaceuticals, pesticides, and dyes, is a toxic, carcinogenic, and persistent compound^6^. One of the most efficient strategies for dealing with NB is reducing NB derivatives to anilines as harmless substances^7–9^. In connection with this, researchers have studied many routes, catalytic systems, and instruments to facilitate the NB derivatives reduction reaction^10,11^^.^

Curcumin (Cur), the primary polyphenol in turmeric, has been utilized as both stabilizing and reducing agent in Au and Ag nanoparticles (NPs) preparation^12^. Recently, Sinha et al. have prepared Cur stabilized AgNPs for the conversion of *p*-nitrophenol to *p*-aminophenol. This reaction occurred under mild conditions without side reactions. However, the attachment of Cur to the metals has some synergistic effect (concerning the catalyst being an electron conduit for reduction of *p*-nitrophenol) to enhance the catalytic active sites number per the catalyst’s unit surface area^13^. Among a large number of efficient nanocatalysts, iron oxide (Fe_3_O_4_ NPs) are highly prized due to their magnetic features, large surface area, convenient surface functionalization, remarkable thermal stability, non-toxic nature, and therapeutic characteristics. Therefore, it is gaining increased attention^14–23^. In this regard, the combination of the magnetic nanoparticles (MNPs) and polymeric materials leads to the formation of a novel organic–inorganic hybrid substances with dual features that render magnetic characteristics with enhanced stability and improved biocompatibility^24–26^. Recently, a heterogeneous catalytic system comprised of poly(p-phenylenediamine)@Fe_3_O_4_ was prepared by applying [HPy][HSO_4_] ionic liquid to effectively synthesize polyhydroquinoline derivatives wit 90–97% yields^27^. Several studies on magnetic catalytic systems have been reported. Moreover, the functionalization of Fe_3_O_4_@Cur nanopowder was proposed to improve the catalytic performance of Fe_3_O_4_@Cur toward NB derivatives reduction. Many agents have been applied for functionalizing catalysts, such as CPTMS, THPP, and APTES. CPTMS has chlorine atoms that match lone electron pairs to metal cations and interact strongly with each other^28^. Melamine (Mel) was attached to the Fe_3_O_4_@Cur@CPTMS through a nucleophilic displacement of the chlorine groups in the CPTMS. For heterogeneous catalysis, selecting a suitable crosslinker is very important as it can influence the succeeding loading rate^2,29–31^. Traditionally, Mel has been well-known and widely used as a suitable crosslinker due to its remarkable chelating capability with metal ions^32,33^. For instance, Nazarzadeh Zare et al. have applied Mel as a crosslinking agent for poly (styrene-co-maleic anhydride). Then, the sulfonated system was magnetized via in situ formation of Fe_3_O_4_ MNPs. This efficient system demonstrated a priviledged performance in synthesis of pyrano[3,2-*c*]chromene, pyrano[2,3-c]pyrazole, and benzylpyrazolyl coumarin^34^. Since Mel has abundant aminal groups, providing rich sites for chelating to metals, Chemical post-modification, occurs conveniently. This ability of Mel has led researchers to develop various catalytic or absorption systems to remove heavy metals from water resources. For example, various Mel-modified polymer systems have been designed to rapidly remove copper (II)^35^, lead (II) and zinc (II)^36^, and methylene blue^37^ from aqueous solutions.

Furthermore, it was hypothesized that porous polymers with Mel linker, possessing many nitrogen atoms, could enhance the Pd immobilization and reduce leaching due to electrostatic interactions ^38^. Albeit several fabrication approaches have been reported, producing AgNPs with both high stability and extended applicability remains a major challenge. Following our previous studies, a PVA-coated iron oxide NPs decorated with silver nanoparticles (Ag NPs) was introduced to reduce the NB derivatives to anilines utilizing hydrazine hydrate (N_2_H_4_.H_2_O)^39,40^.

As a practical approach, the NB derivatives reduction via N_2_H_4_.H_2_O in the presence of nanoscale catalysts were introduced. For example, a method was recently employed by Anbu et al., which was implemented in CeO_2_ NPs^41^. Notably, a large surface area has been developed based on the nanoscale AgNPs incorporated into the structure from the physical aspects. Also, their broad catalytic applicability is referred to as the AgNPs’ efficient electronic and optical characteristics. Based on the previously reported literature and our previous experience, Ag NPs have high surface energy, leading to rapid aggregation^42,43^. Correspondingly, Ag NPs act as an important reducing agent in catalytic systems. Furthermore, immobilization of Ag NPs on polymeric substrates such as chitosan promotes aggregation^8,44^. An efficient in situ synthesis of AgNPs containing polyvinyl alcohol (PVA)-guar gum (GG) composite as PVA-GG-AgNPs was performed to convert NB derivatives to aniline^45^. The NaBH_4_ reducing agent was applied in the NB reduction reaction, which has a role as the hydrogen donator in the aqueous medium. Importantly, this reduction did not carry out in the case of not applying any catalyst according to an expansive potential contrast between the NaBH_4_ (H-donator) and the NB (acceptor), which driven to a kinetic barrier that diminished the practicality of this reaction^46^. Also, the higher Ag electron conductivity led to a local electronic structure slight alternation, which considerably aids in upgrading the catalytic activity related to the mono-metal catalyst^47^.

Herein, we described a synthetic route for preparing magnetic nanocatalyst based on CPTMS functionalization of Fe_3_O_4_@Cur followed by Mel linker displacement with CPTMS’ chlorine group and incorporation of Ag NPs via chelating with the Mel linker. The in situ magnetization of Cur through the co-deposition method resulted in a convenient magnetically separating the magnetic nanocomposite by an external magnet. The magnetic nanocomposite rendered a high surface area according to the nanoscale incorporated NPs, i.e., Fe_3_O_4_ and Ag NPs. Using a small amount of NPs is adequate to obtain high-performance results. The prepared Fe_3_O_4_@Cur/Mel-Ag magnetic nanocomposite was employed as a highly efficient nanocatalyst for the transfer hydrogenation of NB. The NB reduction reaction was accomplished in the N_2_H_4_.H_2_O presence and under mild conditions. Moreover, remarkable reaction yields of 98% were obtained in short-time reactions. Besides, the prepared magnetic nanocatalyst was recycled 5 times, and no significant reduction in catalytic yield was detected.

## Results and discussion

### Preparation of Fe_3_O_4_@Cur/Mel-Ag magnetic nanocomposite

**Scheme 1:**
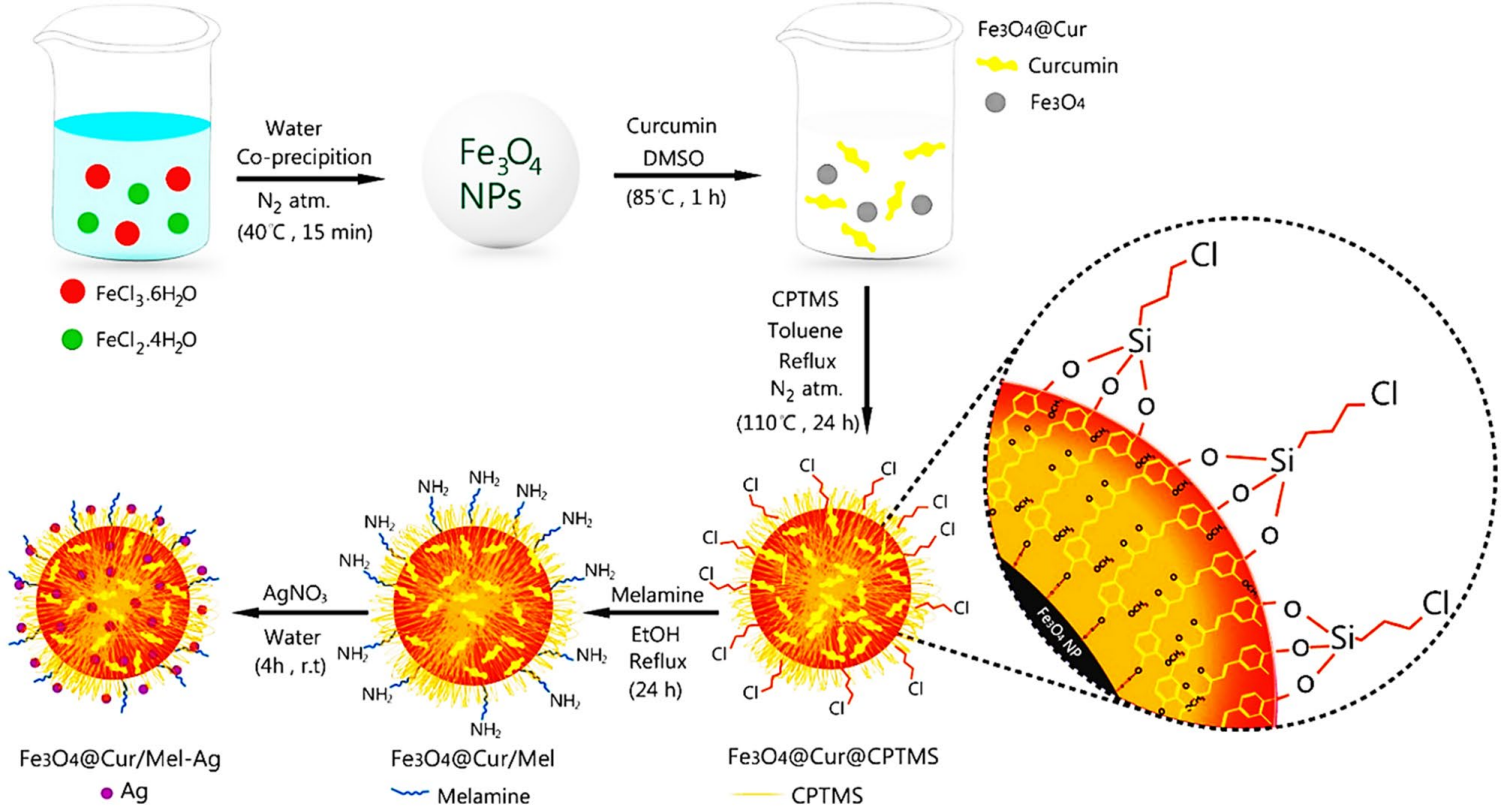
The synthetic approach of the Fe_3_O_4_@Cur/Mel-Ag magnetic nanocomposite.

In this work, the in situ magnetization of Cur with Fe_3_O_4_ superparamagnetic nanoparticles (Fe_3_O_4_@Cur) was carried out through the co-precipitation approach by adding an aqueous mixture of Fe^3+^ and Fe^2+^ salts into the reaction containing Cur solution in DMSO^48^. The mixture was followed by the dropwise addition of ammonia to raise the pH into the basic range. The magnetic curcumin was subsequently functionalized with 3-chloropropyltrimethoxysilane (CPTMS) through an SN2 reaction by removing methoxy groups to prepare Fe_3_O_4_@Cur^49^. Then, the attachment of Mel under reflux conditions and ethanol solution occurred by a substitution reaction (Fe_3_O_4_@Cur/Mel)^50^. The immobilization of the AgNPs in the final stage as the catalytic active sites in nitroarenes reduction was accomplished by stirring the AgNO_3_ salt added to the reaction flask containing Fe_3_O_4_@Cur/Mel magnetic nanocatalyst^10^. Numerous approaches have been previously reported to stabilize and reduce Ag ions to Ag NPs by utilizing various polymers, namely polyvinyl alcohol^51^, polyethylene glycol^52^, and polyvinyl chloride^53^. The preparation route of the Fe_3_O_4_@Cur/Mel-Ag magnetic nanocomposite was depicted in Scheme 1. Eventually, the final dark brown magnetic nanocomposite was characterized by FTIR, EDX, TGA, VSM, and SEM analyses.

### Characterization

#### Fourier-Transform Infrared Spectroscopy

**Figure 1:**
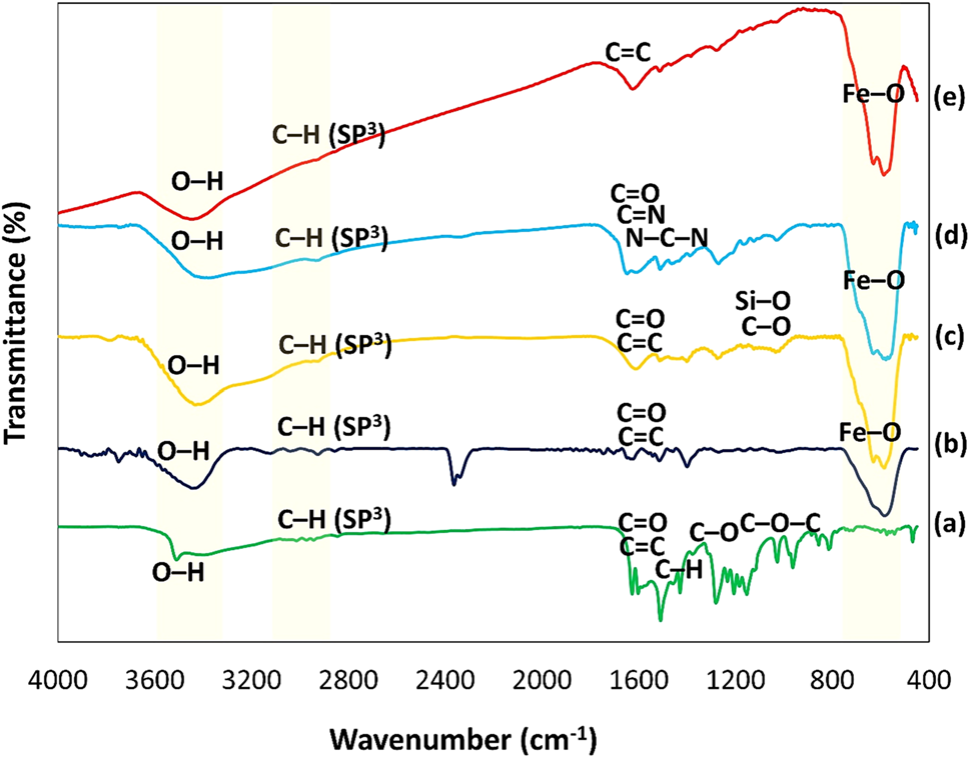
FTIR spectra of (**a**) Cur, (**b**) Fe_3_O_4_@Cur nanopowder, (**c**) Fe_3_O_4_@Cur@CPTMS, (**d**) Fe_3_O_4_@Cur/Mel, (**e**) Fe_3_O_4_@Cur/Mel-Ag magnetic nanocomposite.

The FTIR spectra of Cur, Fe_3_O_4_@Cur, Fe_3_O_4_@Cur@CPTMS, Fe_3_O_4_@Cur/Mel, and Fe_3_O_4_@Cur/Mel-Ag magnetic nanocomposite are plotted in Fig. 1. The distinctive curcumin peaks are shown in Fig. 1a. In this regard, the phenolic O–H stretching vibration, aromatic moiety C = C stretching, benzene ring stretching vibrations, and C = O and C = C vibrations arose at 3508 cm^−1^, 1628 cm^−1^, 1597 cm^−1^, 1509 cm^−1^, respectively. Moreover, the peaks that emerged at 1428 cm^−1^, 1278 cm^−1^, and 1024 cm^−1^ are assigned to the olefinic C-H bending vibrations, aromatic C–O stretching vibrations, and C–O–C stretching vibrations, respectively^54^.

For further interaction corroboration, in the pure curcumin spectrum, a peak at 963 cm^−1^ appeared, which was attributed to the in-plane bending of the enolic section’s hydroxyl group^55^; however, this peak disappeared in the Fe_3_O_4_@Cur, indicating functionalization through the keto-enol functionality in the curcumin polymer. Previous reports have also demonstrated similar interaction, where metal NPs like Au and Ag were functionalized with curcumin through the keto-enol functionality in an aqueous medium^55,56^. The band at 580 cm^−1^, corresponding to the Fe–O stretching vibration, has affirmed the successful incorporation of Fe_3_O_4_ MNPs into the curcumin matrix and appeared in all spectra^57–60^. A peak at ca. 3460 cm^−1^ in the curcumin spectrum displays the –OH presence (Fig. 1b)^23,61,62^. Additionally, due to the Fe_3_O_4_@Cur@CPTMS spectrum in Fig. 1c, the broadband in the 1000–1100 cm^−1^ range was assigned to the C–O and Si–O bonds stretching vibration^49^.

The new peaks that appeared in the FTIR spectrum of Fe_3_O_4_@Cur/Mel (Fig. 1d), at 1625 and 1541 cm^−1^, are ascribed to melamine C = N stretching and N–C–N bending vibrations. According to these bands, successful grafting of melamine onto the Fe_3_O_4_@Cur@CPTMS surface is deduced^50^. As observed in the spectra of Fig. 1e, the intensity of the strong broad peak at 3467 cm^−1^ related to –OH groups, was decreased after the Ag chelation process. In addition, it is deduced that Ag NPs prevent the C–H bond vibrations with sp^3^ hybridation; since the intense decrease in the peak intensity at 2931.0 cm^−1^ was detected. Overall, the peak intensities were diminished after Ag addition to the nanocomposite, and this an approval for Ag loading onto the surface of Fe_3_O_4_@Cur/Mel NPs^10^.

#### Energy-dispersive X-ray spectroscopy

**Figure 2:**
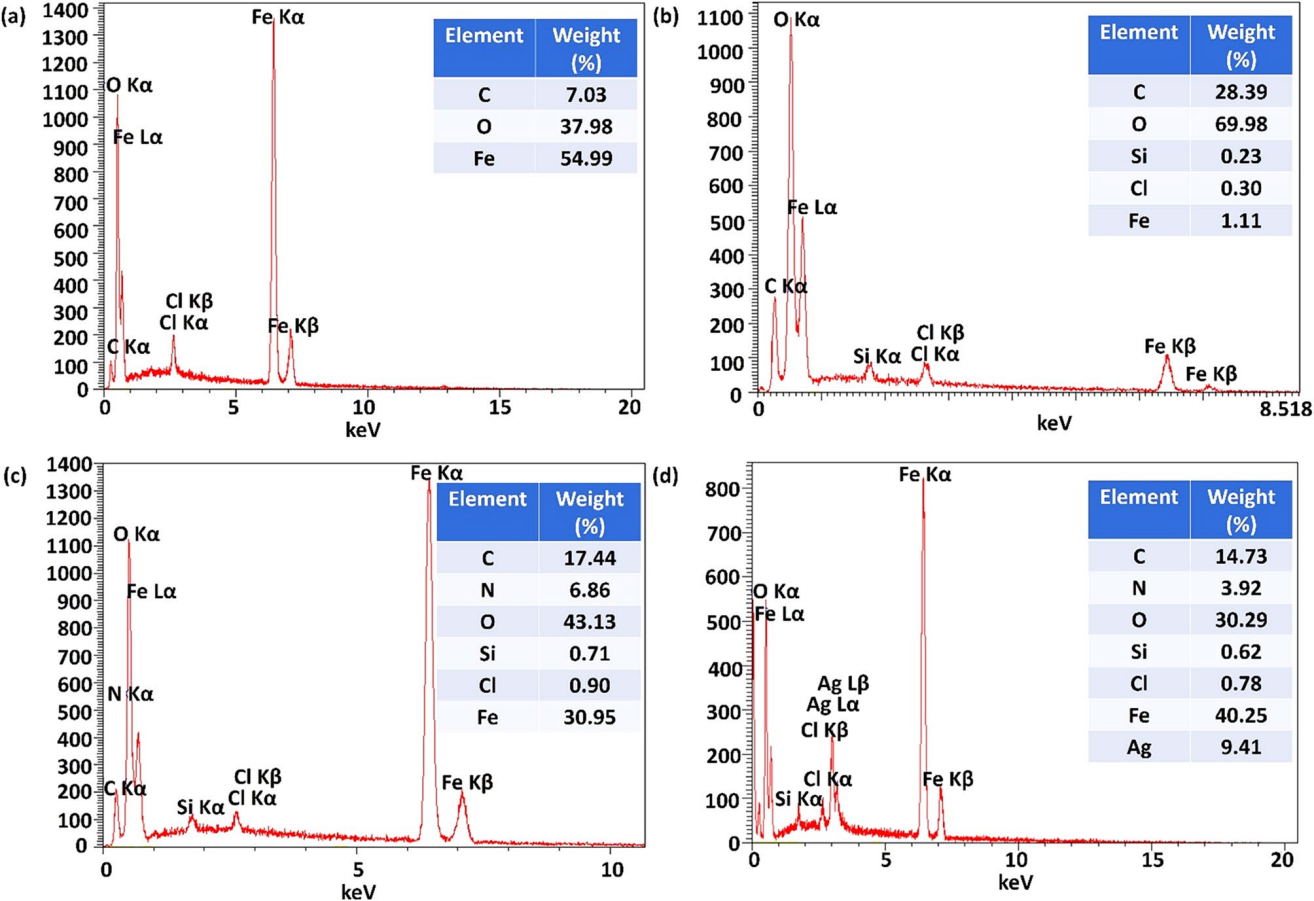
EDX spectra and quantitative table of the structural elements of (**a**) Fe_3_O_4_@Cur nanopowder, (**b**) Fe_3_O_4_@Cur@CPTMS, (**c**) Fe_3_O_4_@Cur/Mel, and (**d**) Fe_3_O_4_@Cur/Mel-Ag magnetic nanocomposite.

The Energy-dispersive X-ray (EDX) spectra of Fe_3_O_4_@Cur nanopowder, Fe_3_O_4_@Cur@CPTMS, Fe_3_O_4_@Cur/Mel, and Fe_3_O_4_@Cur/Mel-Ag magnetic nanocomposite in Fig. 2 confirm the presence of elements in different preparation steps. The carbon and oxygen elements with 7.03 and 37.98 W% demonstrate the presence of Cur in magnetized Fe_3_O_4_@Cur nanopowder (Fig. 2a). According to Fig. 2a, b, the percentage of carbon and oxygen enhanced due to functionalization of magnetic nanocomposite with CPTMS and Mel besides, the existence of Si and Cl with 0.23 and 0.30 W%, respectively, in panel b indicates the successful attachment of CPTMS. Moreover, the effective addition of Mel to Fe_3_O_4_@Cur@CPTMS magnetic nanocomposite can be related to the N signal in panel c with a weight percentage of 0.71. According to the EDS quantitative table in panel d, the Ag NPs as active catalytic sites in nitroarene reduction have been incorporated into the Fe_3_O_4_@Cur/Mel-Ag with a desirable weight percent of 9.42 W%, indicating the successful preparation of the final magnetic nanocomposite.

#### Thermogravimetric analysis

**Figure 3:**
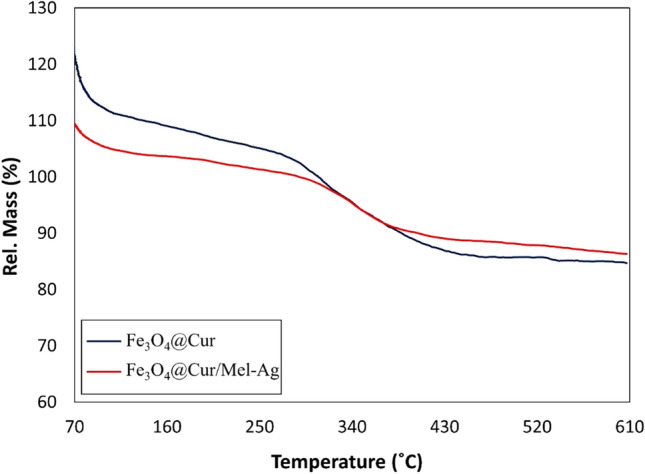
Weight loss versus temperature TGA curves of Fe_3_O_4_@Cur nanopowder and Fe_3_O_4_@Cur/Mel-Ag magnetic nanocomposite, heated up to 610 °C in air.

The thermal stability of Fe_3_O_4_@Cur nanopowder and Fe_3_O_4_@Cur/Mel-Ag magnetic nanocomposite was analyzed by TGA analysis, as shown in Fig. 3. The first stage of mass loss between 70 to about 110 °C was associated with the evaporation of absorbed and bound water in Fe_3_O_4_@Cur nanopowder and Fe_3_O_4_@Cur/Mel-Ag magnetic nanocomposite. Furthermore, the impressive weight loss of Fe_3_O_4_@Cur nanopowder at 280 to 420 °C is due to the degradation of Cur. Also, the improvement of thermal stability (between 300 to 400 °C) in Fe_3_O_4_@Cur/Mel-Ag is related to the interaction between Cur and Mel^63^.

#### XRD patterns

**Figure 4:**
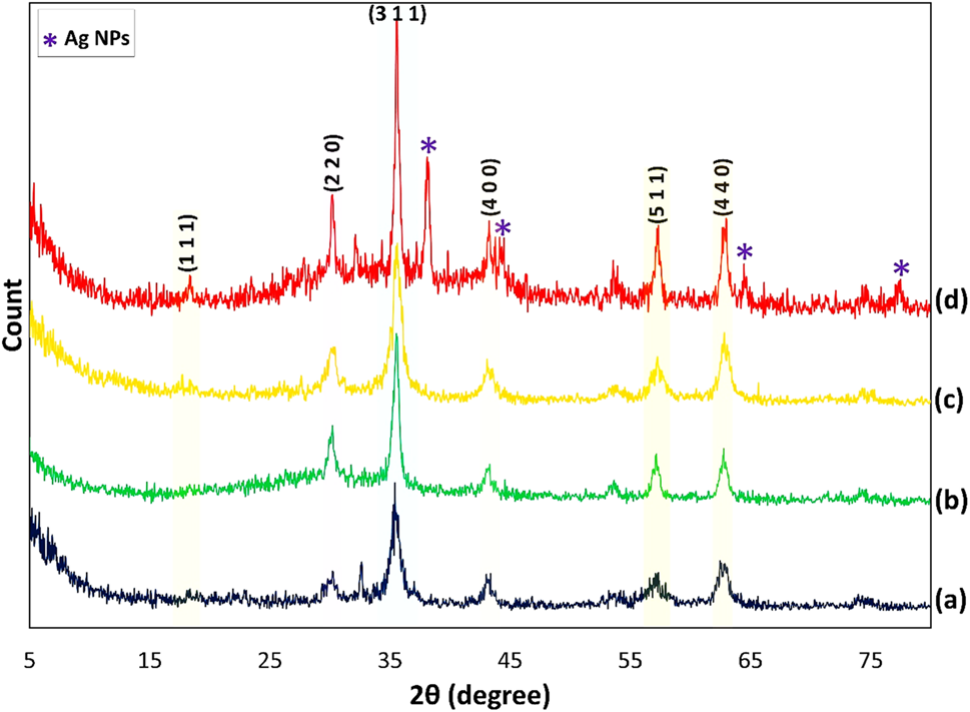
XRD pattern of (**a**) Fe_3_O_4_@Cur nanopowder, (**b**) Fe_3_O_4_@Cur@CPTMS, (**c**) Fe_3_O_4_@Cur/Mel, (**d**) Fe_3_O_4_@Cur/Mel-Ag magnetic nanocomposite.

Figure 4 demonstrates the X-ray diffraction pattern of the Fe_3_O_4_@Cur nanopowder, Fe_3_O_4_@Cur/Mel, and Fe_3_O_4_@Cur/Mel-Ag magnetic nanocomposite. The appeared peaks at 2θ = 18.63, 30.17, 35.54, 43.05, 57.14, and 62.66 are marked by their miller indices (1 1 1), (2 2 0), (3 1 1), (4 0 0), (5 1 1), and (4 4 0) that are corresponded to the Fe_3_O_4_ NPs with 01-088-0315 JCPDS reference code^64,65^. The Cur polymer was applied on the surface of Fe_3_O_4_ magnetic NPs. Therefore, the Cur interaction with different facets on the nanoparticle surface may decrease or increases the specific planes’ growth rate along specific directions, leading to such peak intensity variations from the XRD analysis (Fig. 4a)^61^. The peaks in Fig. 4b at ca. 43° and 60° are related to the functionalization by CPTMS, but they overlap with (4 0 0) and (4 4 0) of miller indices of Fe_3_O_4_ magnetic NPs^66^. The attained results in Fig. 4b–d represent the crystalline phase stability of Fe_3_O_4_ NPs during the modification^50^. Also, the overlapped peaks of the Mel linker with Fe_3_O_4_ NPs have led to increased intensity (Fig. 4c)^67,68^. Furthermore, as shown in Fig. 4d, the distinctive peaks emerged at 2θ = 38.26°, 44.47°, 64.71°, and 77.74° are ascribed to the Ag NPs diffraction pattern with the corresponding 01-087-0719 JCPDS reference code^10^.

#### Electron microscopy imaging

**Figure 5:**
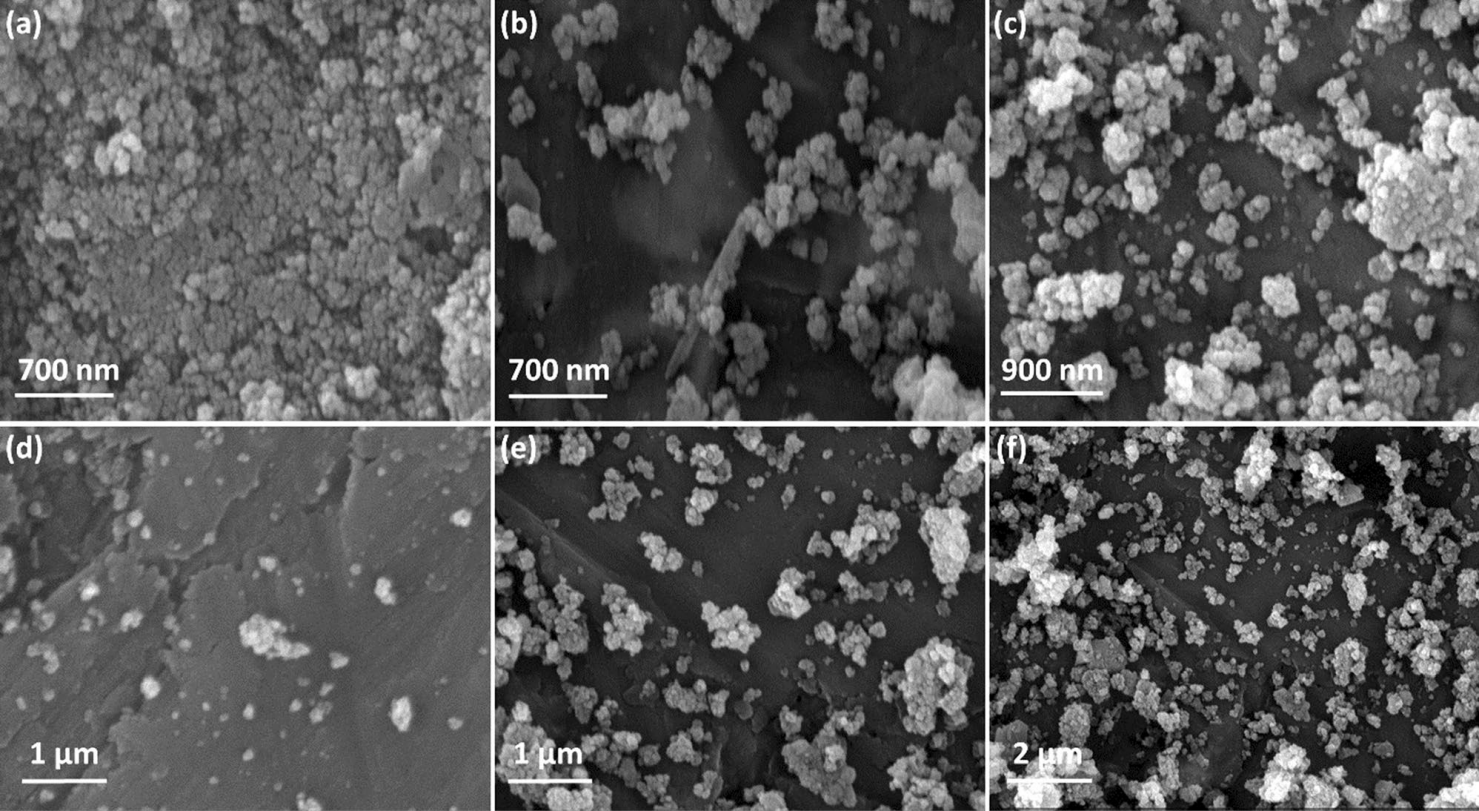
SEM images of (**a**) Fe_3_O_4_@Cur nanopowder, (**b, c**) Fe_3_O_4_@Cur@CPTMS, (**d**) Fe_3_O_4_@Cur/Mel, (**e, f**) Fe_3_O_4_@Cur/Mel-Ag magnetic nanocomposite.

The morphology evaluation of Fe_3_O_4_@Cur nanopowder, Fe_3_O_4_@Cur@CPTMS, Fe_3_O_4_@Cur/Mel, and Fe_3_O_4_@Cur/Mel-Ag magnetic nanocomposite is demonstrated by scanning electron microscope (SEM) images in Fig. 5. After in situ magnetization of Cur by Fe_3_O_4_ MNPs, the prepared superparamagnetic NPs were formed throughout the Cur context with desirable shape uniformity and relatively regular size of ca. 30 nm (Fig. 5a). However, the tendency of individual MNPs to agglomerate results in larger agglomeration. Figure 5b, c revealed the size alteration after functionalizing with CPTMS. That is, the Fe_3_O_4_ MNPs aggregates became larger, and their dispersion over the curcumin polymeric context was not very uniform after functionalization. After the Mel addition, the aggregates were more stuck together, and each aggregate was far from the other. Also, the curcumin polymeric context with a uniform and flat surface can be observed in Fig. 5d. The surface morphology of Fe_3_O_4_@Cur/Mel-Ag magnetic nanocomposite is presented in Fig. 5e, f. The bright spots on the surface of the aggregates are ascribed to the AgNPs. Moreover, chelating the AgNPs to the Mel caused a wider dispersion of the particles throughout the curcumin.

#### Vibrating-sample magnetometer analysis

**Figure 6:**
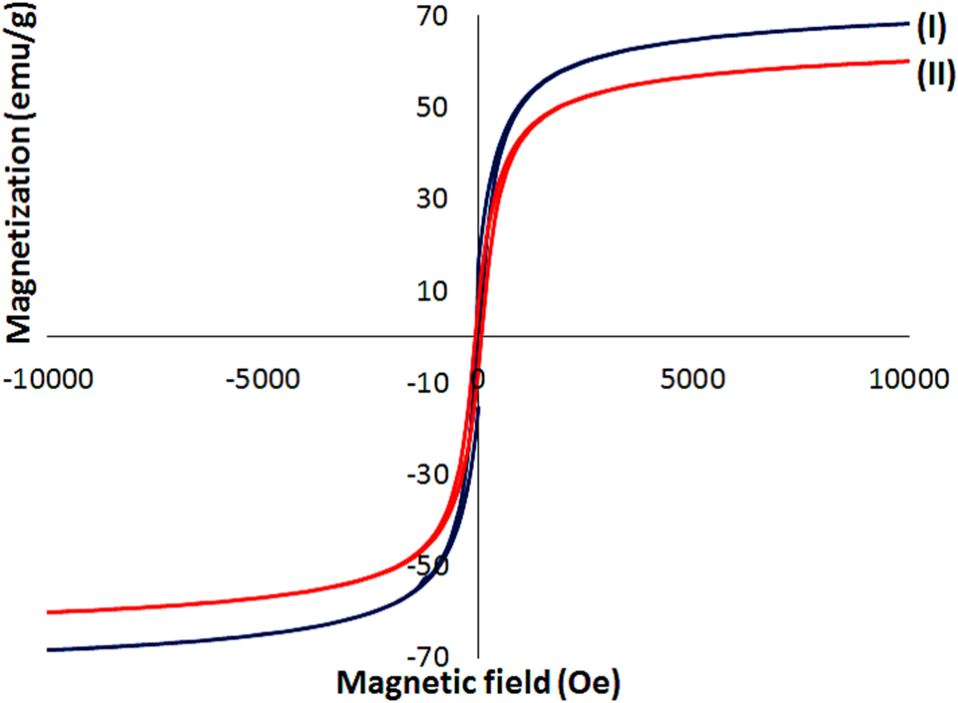
(**a**) The room temperature M–H curves of (**I**) Fe_3_O_4_@Cur nanopowder and (**II**) Fe_3_O_4_@Cur/Mel-Ag magnetic nanocomposite.

Magnetic susceptibility and saturation value of Fe_3_O_4_@Cur nanopowder and Fe_3_O_4_@Cur/Mel-Ag magnetic nanocomposite were determined by vibrating-sample magnetometer (VSM) analysis, as in Fig. 6. The magnetic saturation of bare Fe_3_O_4_ magnetic NPs is reported to be 71 amu g^−1^^69^. However, the reduced magnetic saturation is proportional with the non-magnetic coating layers integrated into the magnetic nanocomposites as it is indicated in Fe_3_O_4_@Cur nanopowder (Fig. 6I) and Fe_3_O_4_@Cur/Mel-Ag magnetic nanocomposite (Fig. 6(II)). Therefore, these nanocomposites’ magnetic saturations have decreased to 68 and 52 emu g^−1^, respectively.

#### The N_2_ adsorption–desorption isotherm

**Figure 7:**
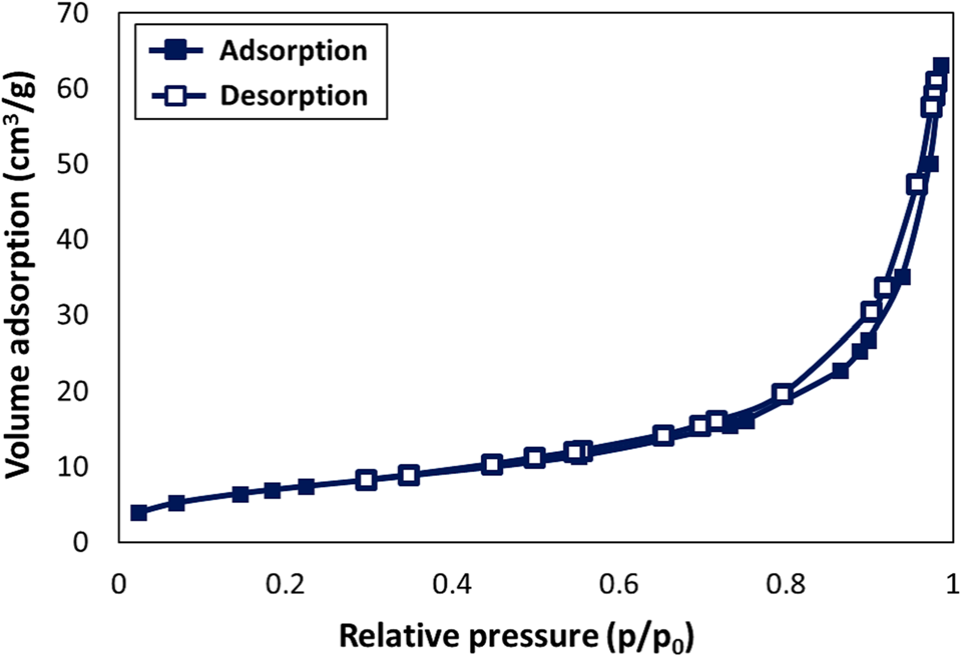
The N_2_ adsorption–desorption isotherms of Fe_3_O_4_@Cur/Mel-Ag magnetic nanocomposite.

The adsorption/desorption of N_2_ gas Brunauer − Emmett − Teller (BET) analysis was applied to evaluate the catalyst’s structural features, surface area and the porosity type. The BET curve of the Fe_3_O_4_@Cur/Mel-Ag magnetic catalytic system is presented in Fig. 7. The BET specific surface area, pore volume, and average pore width of the catalyst were estimated to be 25.778 m^2^/g, 0.097 cm^3^/g, and 15.154 nm^70^. The hysteresis loop of the presented BET analysis represent type IV isotherm of the mesoporous materials with very narrow capillary pores. This state of the mesoporous structures would be appropriate for entrapment of the starting materials into the pores which leads to tight interactions and increasing the probability of materials collision.

### Application

#### Optimizations

To reach the optimized condition, the Fe_3_O_4_@Cur/Mel-Ag magnetic nanocomposite catalytic performance was explored. In this way, various catalytic amounts of the nanocomposite and the applied hydrazine amount in the catalyzed synthesis of the aniline derivatives reaction experimented. The detailed experiment information is rendered in Table 1. As asserted in the table, to clarify the individual nanocomposite’s moieties, such as Fe_3_O_4_ MNPs, the Cur/Mel-Ag was employed in the reduction reaction under the same condition. It is demonstrated that the reaction yield was diminished after Fe_3_O_4_ MNPs removal from the nanocomposite (Table 1, entry 11). Furthermore, since the main catalytic active site of this nanocomposite is AgNPs, it was predictable that removing these NPs from the catalytic system would lead to a significant decrease in the reaction yield (Table 1, entry 2). As claimed by the table, the optimum conditions for NB derivatives reduction reaction were utilizing 0.02 g of Fe_3_O_4_@Cur/Mel-Ag magnetic nanocomposite, 10 min reaction under stirring, and an ambient temperature (Table 1, entry 5).

**Table 1:** Optimization of the NB reduction reaction conditions by applying various amounts of nanocomposite and hydrazine in various solvents and conditions.

Entry	Cat	Cat. ratio (mg)	N_2_H_4_ (mol %)	NaBH_4_ (mmol)	Time (min)	Solvent	Conditions	Yield^a^ (%)
1	–	–	5	–	20	EtOH	–	Trace
2	Fe_3_O_4_@Cur/Mel	20	5	2	10	EtOH	Reflux	25
3	Fe_3_O_4_@Cur/Mel-Ag	5	5	2	10	EtOH	Reflux	80
4	Fe_3_O_4_@Cur/Mel-Ag	10	5	2	10	EtOH	Reflux	93
5	Fe_3_O_4_@Cur/Mel-Ag	20	5	2	10	EtOH	Reflux	98*
6	Fe_3_O_4_@Cur/Mel-Ag	30	5	2	10	EtOH	Reflux	98
7	Fe_3_O_4_@Cur/Mel-Ag	40	5	1	10	EtOH	Reflux	91
8	Fe_3_O_4_@Cur/Mel-Ag	20	4	3	10	EtOH	Reflux	93
9	Fe_3_O_4_@Cur/Mel-Ag	20	2	2	10	EtOH	Reflux	81
10	Fe_3_O_4_@Cur/Mel-Ag	20	1	2	10	EtOH	Reflux	74
11	Cur/Mel-Ag	20	5	2	10	EtOH	Reflux	95
12	Fe_3_O_4_@Cur/Mel	10	1	2	50	EtOH	Reflux	Trace
13	Fe_3_O_4_@Cur/Mel-Ag	10	5	2	10	EtOH	r.t	71
14	Fe_3_O_4_@Cur/Mel-Ag	10	5	1	10	MeOH	Reflux	88
15	Fe_3_O_4_@Cur/Mel-Ag	5	5	3	10	H_2_O	Reflux	79
16	Fe_3_O_4_@Cur/Mel-Ag	20	5	2	10	CH_3_CN	Reflux	91
17	Fe_3_O_4_@Cur/Mel-Ag	20	4	2	10	PEG-400	Reflux	93
18	Fe_3_O_4_@Cur/Mel-Ag	10	2	2	10	CH_2_Cl_2_	Reflux	53

^a^Isolated yields.

*Opimized conditions.

All the optimization reactions were implemented under reflux conditions. The progress rate of catalyzed reduction reactions was perused by thin-layer chromatography (TLC). The resulting aniline derivative products have been proved by FT-IR spectroscopy and melting points. The efficiency of AgNPs as main catalytic active sites was also considered by comparing catalytic performance of the Fe_3_O_4_@Cur/Mel and Fe_3_O_4_@Cur/Mel-Ag magnetic nanocomposite in the NB reduction reaction (Table 1). Based on Table 1, the highest yield has been achieved in the case of applying 0.02 g of Fe_3_O_4_@Cur/Mel-Ag in the presence of (5 mol%) hydrazine hydrate in ethanol during a 10 min reaction time.

#### Catalytic activity

First, to evaluate the prepared Fe_3_O_4_@Cur/Mel-Ag magnetic nanocomposite catalytic efficiency, different reaction variables, such as solvents, catalytic ratios, and hydrazine amounts in NB reduction reaction, were optimized (Table 2, entry 1). Extensive information on the optimization procedure has been presented in Table 1. After that, the catalytic activity of the Fe_3_O_4_@Cur/Mel-Ag magnetic nanocomposite was investigated by applying different NB derivatives, as represented in Table 1. As shown in Table 1, the high reaction yields were achieved in a short reaction time. Nonetheless, these outcomes affirm the Fe_3_O_4_@Cur/Mel-Ag magnetic nanocomposite’s high catalytic activity compared with the previously reported catalysts, as shown in Table 3. The Fe_3_O_4_@Cur/Mel-Ag can be assumed to be a significant catalyst in NB reduction reactions. For catalytic performance investigation of the Fe_3_O_4_ MNPs, Cur/Mel-Ag was used under the same conditions. Due to Table 1, entry 11, a partial decrease in the attained catalytic yields after the Fe_3_O_4_ MNPs removal was observed. Additionally, the ^1^H-NMR and ^13^C-NMR spectra and spectral data of aniline and its derivatives are presented in the supporting information file (Figures S1–S10).

**Table 2:**
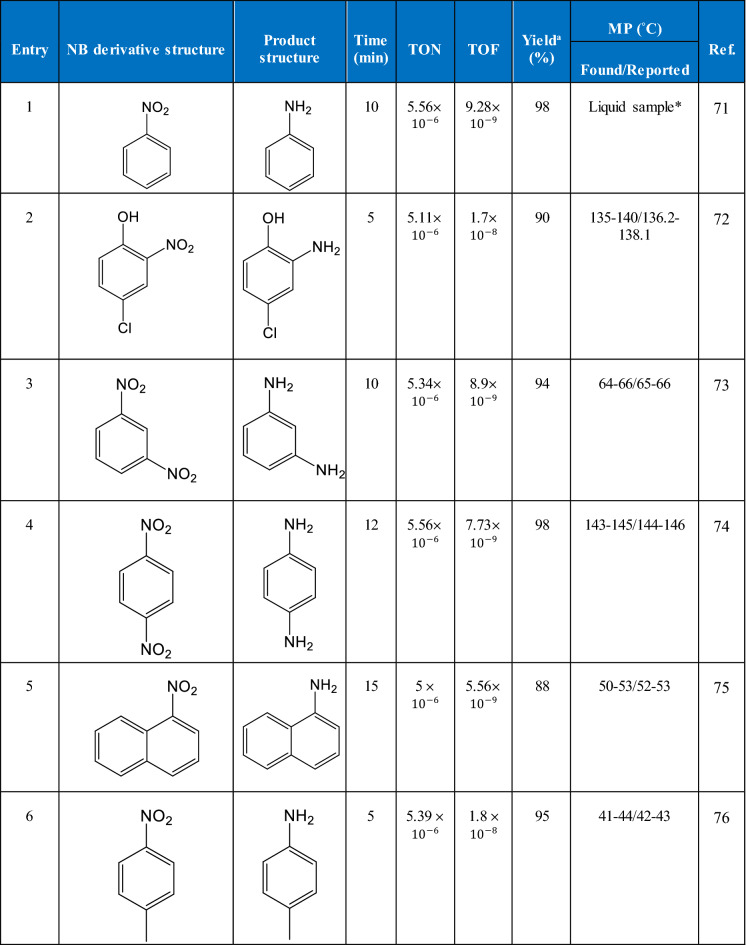

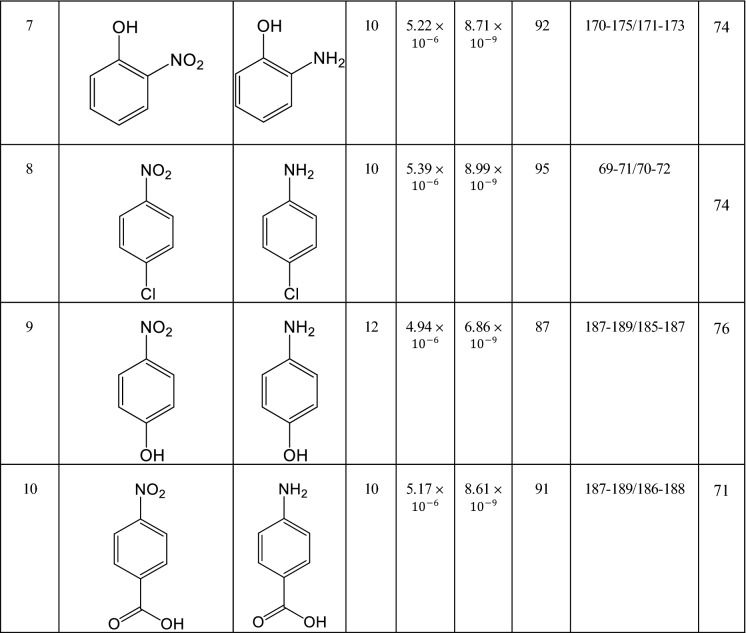
The obtained yields after NB derivatives reduction to aniline by applying Fe_3_O_4_@Cur/Mel-Ag magnetic nanocomposite 0.02 g, NB 1.0 mmol, N_2_H_4_.H_2_O 5 mol%, and ethanol 2.0 mL, at 70 °C.

*Liquid sample was identified with boiling point screening and thin-layer chromatography (TLC) (bp ca.184 °C)

^a^The yields referred to the isolated products.

**Table 3:** Comparison of the obtained results in this study with some other reported catalysts.

Entry	Catalyst	Condition	Time (min)	Yield (%)	Ref
1	Ag@P (SNA-CS) (57.5 μg/mL)	N_2_H_4_.H_2_O (10.95 mmol)/28 ± 1 °C	27	94.31	79
2	Fe_3_O_4_‐Glu‐AgNPs (60 mg)	NaBH_4_ (4.0 mmol)/H_2_O, 60 °C	9	98	80
3	TiO_2_@Ag_A	Citric acid:catalyst weight ratio (1.89:1)/25 °C	0.67	100	81
4	Ag-PNA-BIS-2	NaBH_4_ (2.0 mmol)/H_2_O/25 °C	180	97	39
5	Fe_3_O_4_–Ni MNPs	Glycerol/KOH (2 mmol)/80 °C	180	94	82
6	Ag NPs@CMC-AG-Pct (1.5 mmol)	NaBH_4_ (5.0 mmol)/ethanol:water (v/v 1:1)	5	99	83
7	Ag-rGO/g-C_3_N_4_	MeOH	240	99	84
8	Fe_3_O_4_@Cur/Mel-Ag (0.02 g)	NaBH_4_ (2.0 mmol)/0.04 g K_2_CO_3_/pH (8.0)/70 °C	10	98	This work

#### Suggested mechanism

**Figure 8:**
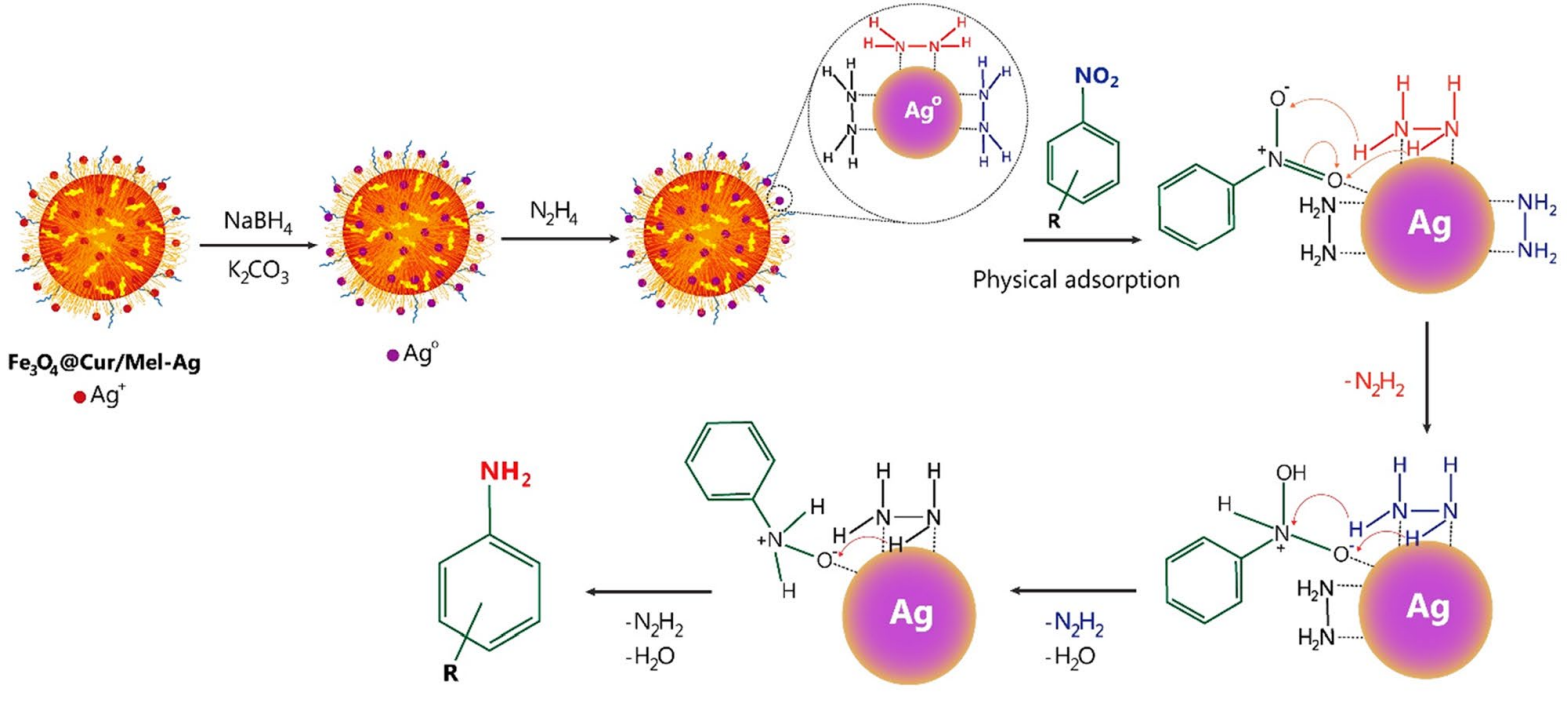
The plausible reaction mechanism of NB derivatives reduction to anilines by applying the Fe_3_O_4_@Cur/Mel-Ag magnetic nanocomposite in the presence of N_2_H_4_.H_2_O.

The plausible mechanism of NB derivatives reduction reaction to aniline derivatives in hydrazine hydrate presence is depicted in Fig. 8, in which the AgNPs act as the main catalytic active sites of the catalytic system. At the initial stage, the Ag^+^ ions as the main catalytic active sites were reduced to Ag^0^ by sodium borohydride (NaBH_4_) under the provided alkaline condition by potassium carbonate (K_2_CO_3_)^11,77,78^. Due to the Figure, with respect to the obtained results in this study and the knowledge from the previous records, it is claimed that efficient electronic interactions between AgNPs and heteroatoms provide a suitable substrate for this reaction type^6^. The heteroatoms’ electronic interactions with hydrazine hydrate’s dissociated hydrogen atoms onto the AgNPs’ surface led to NB derivatives absorption and conversion into anilines during successive dehydration procedures. Hydrazine hydrate has the action of an essential H-supporter for the reduction procedure, which interacts with the surface of AgNPs from its nitrogen sites effectively. Sodium borohydride generally undergoes decomposition in acidic media but not in basic media. In alkaline conditions, sodium metaborate (NaB(OH)_4_) is formed after the NB reduction reaction completion^11^. Ultimately, the aniline derivative structures are produced and left the catalyst's surface, and the particles are magnetically collected from the mixture, rinsed, and recycled several times.

#### Recyclability

**Figure 9:**
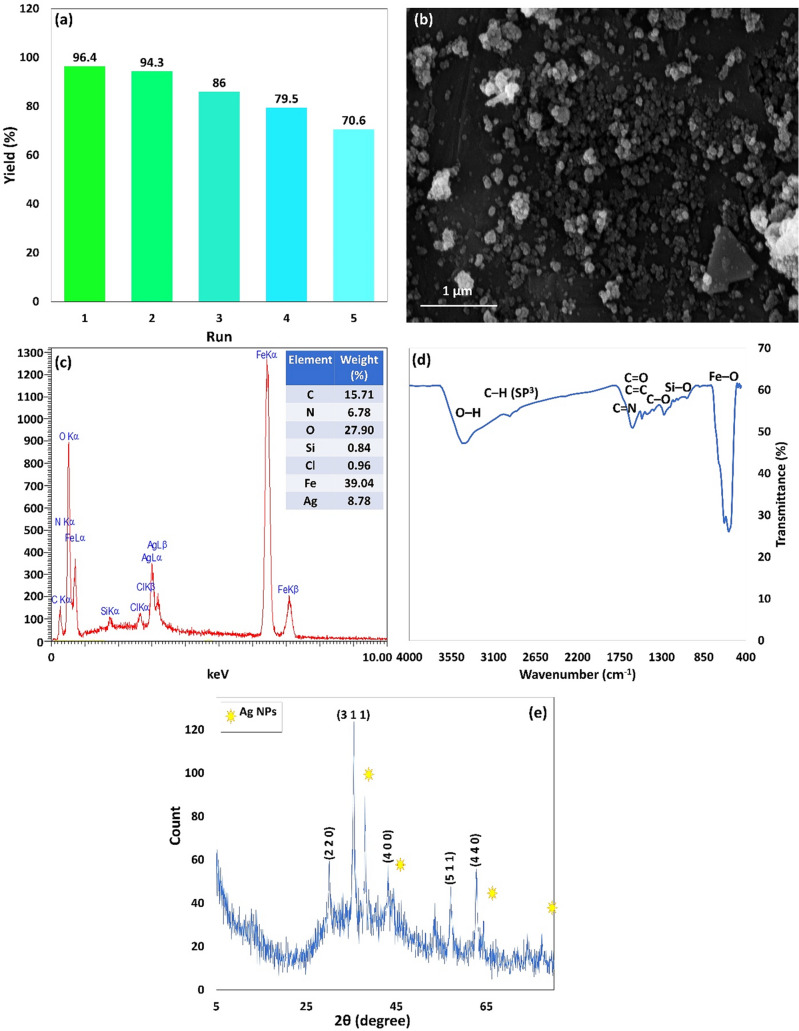
(**a**) The retrievability assessment and (**b**) SEM image, (**c**) EDX, (**d**) FTIR spectrum, and (**e**) XRD pattern of Fe_3_O_4_@Cur/Mel-Ag magnetic nanocomposite after 5 cycles of catalyzed NB reduction reactions.

To evaluate the retrievability of the prepared Fe_3_O_4_@Cur/Mel-Ag magnetic nanocomposite, it was magnetically collected from the NB hydrogenation reaction by an external magnet. The collected catalyst was rinsed with ethanol and deionized water, dried, and reutilized in successive reactions. In the next stage, the particles’ dispersion in deionized water occurred via ultrasonication and was followed by rinsing with ethanol. Eventually, the particles were dried at 60 °C and reutilized in 5 reduction reaction cycles. The observed decrease from 96.4% to 70.6% in Fig. 9a may be attributed to the AgNPs separation during the catalytic procedure. Also, the SEM image of the Fe_3_O_4_@Cur/Mel-Ag magnetic nanocomposite that is presented in Fig. 9b confirmed preservation of the structure of catalytic substrate and the Ag NPs during the catalytic reaction, as the solidity, spherical morphology, and dispersion of the particles are maintained after the recovery. According to the EDX chart as well as its quantitative table (Fig. 9c), the active catalytic sites of the Fe_3_O_4_@Cur/Mel-Ag nanocomposite (Ag nanoparticles) in NB reduction reaction (in optimum reaction condition) remained in the structure of the nanocomposite with 8.78 W% even after five reusing runs. Although the weight percentage of Ag nanoparticles has been reduced from 9.42 W% for the Fe_3_O_4_@Cur/Mel-Ag nanocomposite before the reaction to 8.78 W% after five retrievability cycles, this amount of the remaining Ag nanoparticles in the structure still shows the catalyst’s stability over reaction conditions.

Based on the FTIR spectrum provided from the separated, washed, and dried nanocatalyst after five reusing cycles (Fig. 9d), the presence of the functional groups indicates the catalyst’s structural stability. In this regard, the strong peak at 3406 cm^−1^ is assigned to the O–H stretching vibration^23,61,62^. Also, C=C stretching vibrations of aromatic compounds, C–O stretching vibrations, and C=O and C=C vibrations appeared at 1628 cm^−1^, 1278 cm^−1^, and 1509 cm^−1^, respectively^54^. Fe–O stretching vibration arose with a sharp band at 580 cm^−1^, affirming the proper Fe_3_O_4_ nanoparticles incorporation in the curcumin substrate^57–60^. Based on the CPTMS addition to the nanocomposite in the functionalization stage, the peak at 1000–1100 cm^−1^ is attributed to the stretching vibrations of C–O and Si–O^49^. The observed peaks at 1625 and 1541 cm^−1^ are related to the C=N stretching and N–C–N bending vibrations of the melamine grafted onto the Fe_3_O_4_@Cur@CPTMS surface^50^. However, similar to the FTIR spectrum of the Fe_3_O_4_@Cur/Mel-Ag nanocomposite before the catalytic reaction, the chelation of Ag nanoparticles is confirmed via the decreased intensity of the O–H stretching vibration at 3406 cm^−1^. On the other hand, the intensity reduction at 2931.0 cm^−1^ assigned to the vibrations of the C─H bond with sp^3^ hybridation, can be associated with the Ag nanoparticles^10^. To confirm that the crystallinity of the structure remains unchanged after five sequential reusing cycles, the XRD analysis was carried out from the Fe_3_O_4_@Cur/Mel-Ag magnetic nanocomposite after five recycling runs, and the result is represented in Fig. 9e. The distinctive peaks at 2θ = 30.17, 35.54, 43.05, 57.14, and 62.66 are corroborated with the (2 2 0), (3 1 1), (4 0 0), (5 1 1), and (4 4 0) miller indices which are related to the 01–088-0315 JCPDS reference code^64,65^. The Cur polymeric network has affected the peak intensity of the prepared Fe_3_O_4_ NPs because of covering the magnetic NPs^61^. Also, CPTMS functionalization appeared with peaks at 43° and 60°. However, these peaks and those related to the Mel linker overlapped with magnetic NPs’ distinguished peaks^66–68^. The Ag NPs with diffraction peaks at 2θ = 38.26°, 44.47°, 64.71°, and 77.74° with 01-087-0719 reference card number have emerged in the XRD pattern^10^.

After the NB reduction completion at optimum conditions, the nanocatalyst was filtered, and the ICP test was taken from the supernatant (Figure S12). Due to the ICP results, the concentration of the Fe^3+^ and Ag^+^ ions released into the supernatant solution was 3.318 ppm and 20.573 ppm, respectively. These release amounts are assigned to the incomplete magnetic nanocatalyst isolation from the reaction mixture after completing the catalytic reaction with a magnet. Thus, the leaching percentage of the Fe_3_O_4_@Cur/Mel-Ag magnetic nanocatalyst or the Fe^3+^ and Ag^+^ ions released into the solution during the catalytic reaction was trivial, and the nanocatalyst can be utilized many times without significant diminish in the adsorption efficiency, which is confirmed with the reusability experiments after five consecutive cycles.

To perform the hot test filtration, as depicted in Figure S13a, the desired solvent (distilled water) was first brought to the boiling temperature (100 °C). Then, 10.0 mL of the boiled solvent was added to the Fe_3_O_4_@Cur/Mel-Ag catalyst Figure S13b, and after some time, the reaction reached the ambient temperature and was filtered with filtration paper. This way, the filtration paper was first washed with boiling water, then the reaction mixture was filtered Figure S13c, and the ICP test was taken from the supernatant. The ICP-OES analysis was executed to determine the amount of Fe^3+^ and Ag^+^ ions released into the supernatant solution at optimum reaction conditions. Based on the obtained results from the ICP analysis, the concentration of the Fe^3+^ and Ag^+^ ions released into the supernatant solution was 0.203 ppm and 0.271 ppm, respectively. This amount can be attributed to the incomplete separation of the magnetic nanocatalyst from the reaction mixture after catalytic reaction completion with an external magnet. Hence, the leaching percentage of the Fe_3_O_4_@Cur/Mel-Ag magnetic nanocatalyst or the Fe^3+^ and Ag^+^ ions released to the solution during the catalytic reaction procedure was negligible, so it could be used several times with no remarkable adsorption efficiency decrease, as affirmed by retrievability experiments.

### Comparisons

In this part, we briefly compare the presented nanocatalysts with some earlier studied systems involving Ag NPs used to convert NBs to aniline analogues. As shown in Table 3, the prepared Fe_3_O_4_@Cur/Mel-Ag magnetic nanocomposite has several superiorities compared to other catalysts. The tendency to use magnetic nanocatalysts is very high as they can easily be separated from the reaction. As explained in the previous section, Fe_3_O_4_ MNPs possess superparamagnetic properties. A high yield of NB derivatives of 98% was obtained at a short reaction time (10 min), while in most cases, the reaction time took more than 2 h (Table 3, entry 4, 5, 7). It should be taken into account that the utilization of inexpensive materials that are highly biocompatible and biodegradable has a high significance in the preparation stages. The incorporation of Cur biopolymer as a substance with a natural source has been preferred according to the economic privileges and its biocompatibility, although other reported catalytic systems have rendered great results (Table 3, entry 3). In addition to the above benefits, the gelish nature of the Cur allows the catalyst particles to be well dispersed, resulting in better overall performance.

## Experimental

### Materials and instruments

All of the chemicals and devices utilized in this study are reported in Tables 4 and 5.

**Table 4:** The name, brand and purity of the used chemical materials in the current work.

Materials	Brand and purity
FeCl_3_·6H_2_O	Merck (Code No. 10025-77-1)
FeCl_2_·4H_2_O	Merck (Code No. 13478-10-9)
Curcumin	Sigma Aldrich (Code No. 458-37-7)
Melamine	Sigma Aldrich (99.0%)
AgNO_3_	Sigma Aldrich (≥ 99.0%)
Ethanol	Sigma Aldrich (96.0%)
Dimethyl sulfoxide (DMSO)	Sigma Aldrich (≥ 99.0%)
Ammonia	Merck (25.0%)
Anhydrous toluene	Sigma Aldrich (99.8%)
3-Chloropropyltrimethoxysilane (CPTMS)	Sigma Aldrich (≥ 97.0%)

**Table 5:** The name and model of the used equipment in the current work.

Instrument	Brand and model
FTIR spectroscopy	Shimadzu FT-IR-8400S
EDX spectroscopy	VEGA-TESCAN-XMU
TGA analysis	Bahr-STA 504
NMR analysis	Varian Unity Inova 500 MHz
XRD	DRON-8 X-ray diffractometer
FESEM	Zeiss Sigma
VSM	Kavir’s LBKFB (Kashan, Iran)
BET	Micromeritics ASAP 2010 device
ICP	Varian VIST-MPX, Axial type: torch
Ultrasonic cleaning bath	Steelco US 80
Oven	Genlab Ltd

### Practical approaches

#### *Preparation of Fe*_*3*_*O*_*4*_*@Cur nano powder*

Concisely, 1.0 g FeCl_2_.4H_2_O and 2.5 g FeCl_3_.6H_2_O were dissolved in 50.0 mL deionized water under constant N_2_ flow at 40 °C for 15 min. Next, 2.0 mg Cur was dissolved in 500.0 μL of DMSO and dropwise added to the reaction mixture. The temperature was elevated to 85 °C; then, ammonia (25.0%, 5.0 mL) was poured into the reaction flask under vigorous stirring of the solution for 1 h. The mixture was rinsed several times using deionized water and dried at 60 °C^85^.

#### *Preparation of Fe*_*3*_*O*_*4*_*@Cur@CPTMS*

First, 1.0 g Fe_3_O_4_@Cur was added into a round bottom flask containing 20.0 mL of dry toluene. The reaction mixture was sonicated in the ultrasound bath for 20 min. For organic modification of the magnetic nanoparticles (MNPs) with 3-chloropropyltrimethoxysilane (CPTMS), 3.0 mL of CPTMS (16.45 mmol) was added to the stirred mixture and refluxed in toluene at 110 °C for 24 h, under N_2_ atmosphere. Afterward, the resultant was rinsed with absolute ethanol for unattached substrate removal and ultimately dried at 100 °C for 12 h to provide Fe_3_O_4_@Cur@CPTMS^49^_._

#### *Preparation of Fe*_*3*_*O*_*4*_*@Cur/Mel*

For Mel-functionalization of the Fe_3_O_4_@Cur, 1.0 g of the as-prepared Fe_3_O_4_@Cur was added to 50.0 mL EtOH; then, Mel (0.13 g, 1.0 mmol) was poured, and the stirred mixture was refluxed for 24 h. The resultant solid (Fe_3_O_4_@Cur/Mel) was magnetically collected and rinsed with EtOH and H_2_O several times and dried at an ambient temperature^50^.

#### *Preparation of Fe*_*3*_*O*_*4*_*@Cur/Mel-Ag magnetic nanocomposite*

0.5 g of the prepared Fe_3_O_4_@Cur/Mel was dispersed well in 15.0 mL of deionized water by applying an ultrasonic bath. Then, AgNO_3_ (1.0 g, 5.9 mmol) was poured into the reaction flask under stirring for 4 h at an ambient temperature. Afterward, the Ag^+^ ions as the main catalytic active sites were reduced to Ag^0^ by sodium borohydride (NaBH_4_) in an alkaline medium provided by potassium carbonate (K_2_CO_3_)^11,77,78^. Finally, the Fe_3_O_4_@Cur/Mel-Ag magnetic nanocomposite was separated using an external magnet and rinsed several times with ethanol and deionized water. The dark brown magnetic nanocomposite dried at 60 °C^10^.

### General procedure for catalytic reduction of nitroarenes by Fe_3_O_4_@Cur/Mel-Ag magnetic nanocomposite

To a 25.0 mL round-bottom flask containing 5.0 mL deionized water, 1.0 mmol, 0.123 g nitrobenzene, and 0.02 g Fe_3_O_4_@Cur/Mel-Ag magnetic nanocomposite were added. Afterward, the pH of the mixture was adjusted to about 8.0 by 0.04 g potassium carbonate addition. Then, 5.0 mol% sodium borohydride was poured into the reaction mixture, and the mixture was exposed to stirring at 70 °C. After the reaction completion, the catalyst was magnetically collected, and the extraction of the product was carried out by EtOAc. Drying the organic layer was accomplished over anhydrous sodium sulfate. The pure aniline with 98% yield was provided by Evaporating the solvent under reduced pressure.

## Conclusion

Among various harmful chemical substances, NB derivatives are chemical compounds that must be eliminated or converted to non-toxic aniline derivatives by appropriate strategies. In this way, one of the most effective approaches is to utilize magnetic nanocatalysts because magnetic nanocatalysts possess excellent characteristics, namely magnetic properties, extreme surface area, surface functionalization ability, excellent thermal stability, and non-toxicity. Here, an efficient heterogeneous magnetic nanocomposite (Fe_3_O_4_@Cur/Mel-Ag) comprised of Fe_3_O_4_ MNPs, Cur biopolymer strands was prepared, followed by functionalization through CPTMS and melamine, and chelating to Ag NPs as catalytic active sites for the conversion of NB derivatives to the aniline form. Based on the proposed mechanism, AgNPs loaded on the surface of Fe_3_O_4_@Cur/Mel are involved in this nanocomposite’s catalytic activity through electronic interactions with heteroatoms. All structural properties analyses, including FTIR, EDX, VSM, XRD, and TGA, have been performed, and the attained results have been debated in context. Besides, a high reaction yield of 98% has been obtained in a 10-min short-time reaction. The Fe_3_O_4_@Cur/Mel-Ag magnetic nanocomposite was separated easily by an external magnet and reused 5 times with no considerable catalytic performance reduction.

## Supplementary Information

Supplementary Information.

## Data Availability

The datasets used and/or analysed during the current study available from the corresponding author on reasonable request.
