# Supplementary material for: Silver-assisted reduction of nitroarenes by an Ag-embedded curcumin/melamine-functionalized magnetic nanocatalyst
Source: Sci Rep. 2023 Mar 30;13:5225. doi: 10.1038/s41598-023-32560-1 (PMC10063568; doi:10.1038/s41598-023-32560-1)
Supplement: Supplementary file 1 — Supplementary Information. [file 41598_2023_32560_MOESM1_ESM.docx]

**Silver-Assisted Reduction of Nitroarenes by an Ag-Embedded Curcumin/Melamine-Functionalized Magnetic Nanocatalyst**

Nima Khaleghi, Mohadeseh Forouzandeh-Malati, Fatemeh Ganjali, Zahra Rashvandi, Simindokht Zarei-Shokat, Reza Taheri-Ledari*, Ali Maleki*

Catalysts and Organic Synthesis Research Laboratory, Department of Chemistry, Iran University of Science and Technology, Tehran16846-13114, Iran.

*Corresponding authors. R. Taheri-Ledari, E-mail address: [Rezataheri13661206@gmail.com](mailto:Rezataheri13661206@gmail.com), R_taheri94@alumni.iust.ac.ir; A. Maleki: E-mail address: maleki@iust.ac.ir, Tel.: +98 21 77240640-50; fax: +98 2173021584.


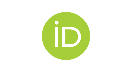
*Author’s ORCID numbers:*

*Reza Taheri-Ledari:* [*https://orcid.org/0000-0002-6511-9411*](https://orcid.org/0000-0002-6511-9411)

*Ali Maleki:* [*https://orcid.org/0000-0001-5490-3350*](https://orcid.org/0000-0001-5490-3350)

***Table of content***

| **Content** | **Page** |
| --- | --- |
| **Figure S1.** ^1^H-NMR and ^13^C-NMR spectra and spectral data of aniline | **S2** |
| **Figure S2.** ^1^H-NMR and ^13^C-NMR spectra and spectral data of 2-amino-4-chloro-phenol | **S3** |
| **Figure S3.** ^1^H-NMR and ^13^C-NMR spectra and spectral data of benzene-1,3-diamine | **S4** |
| **Figure S4.** ^1^H-NMR and ^13^C-NMR spectra and spectral data of benzene-1,4-diamine | **S5** |
| **Figure S5.** ^1^H-NMR and ^13^C-NMR spectra and spectral data of 2-Naphthylamine | **S6** |
| **Figure S6.** ^1^H-NMR and ^13^C-NMR spectra and spectral data of p-toluidine | **S7** |
| **Figure S7.** ^1^H-NMR and ^13^C-NMR spectra and spectral data of 2-aminophenol | **S8** |
| **Figure S8.** ^1^H-NMR and ^13^C-NMR spectra and spectral data of 4-chloroaniline | **S9** |
| **Figure S9.** ^1^H-NMR and ^13^C-NMR spectra and spectral data of 4-amino-phenol | **S10** |
| **Figure S10.** ^1^H-NMR and ^13^C-NMR spectra and spectral data of 4-amino-benzonic acid | **S11** |
| **Figure S11.** The Number-frequency histogram of Fe_3_O_4_ and Fe_3_O_4_@Cur/Mel-Ag magnetic nanocomposite. | **S12** |
| **Figure S12.** Filtered Fe_3_O_4_@Cur/Mel-Ag nanocatalyst solution after the NB reduction reaction | **S13** |
| **Figure S13.** (a) The boiling solvent, (b) The catalyst containing solvent, (c) The filtered solution. | **S14** |

*NOTE: All synthesized aniline derivatives were checked by TLC and melting point measurement. The reported NMR spectra were as-prepared and re-produced from our previous work: DOI: 10.1016/j.catcom.2019.105850.*


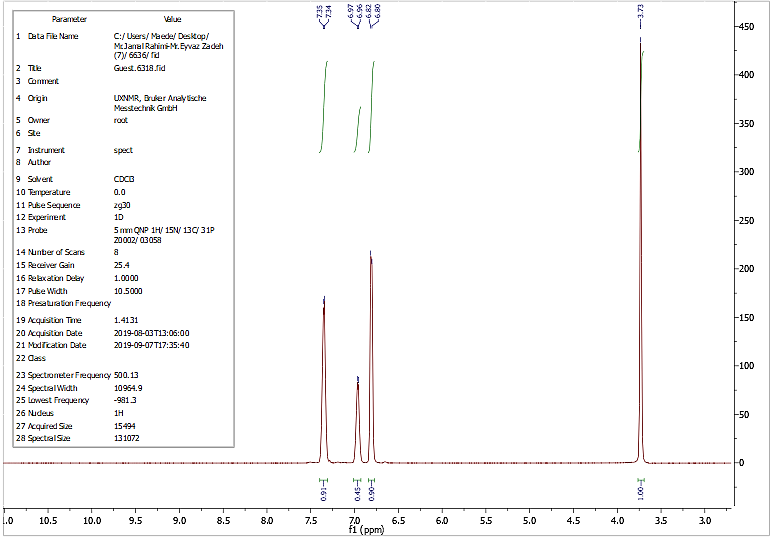


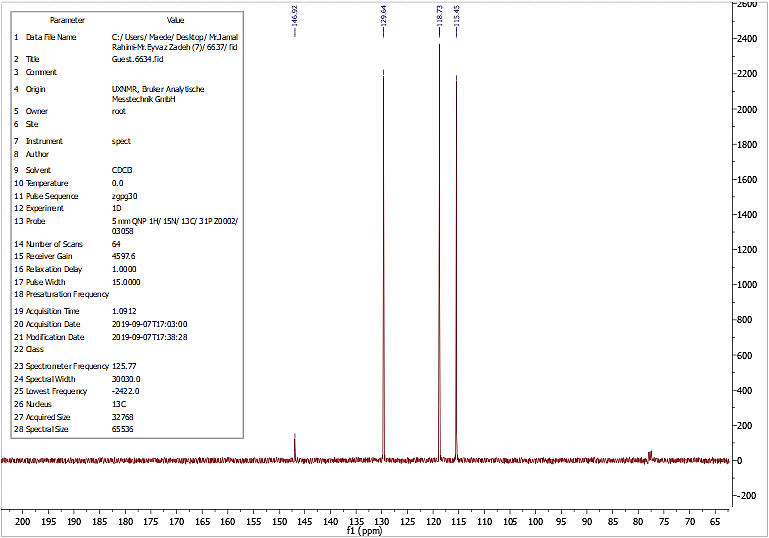


**Figure S1.** ^1^H-NMR and ^13^C-NMR spectra and spectral data of aniline.

Sepectral data: ^1^H NMR (500 MHz, CDCl_3_) δ 3.73 (s, 2 H), 6.80-6.82 (d, J = 10 Hz, 2 H), 6.93-6.99(m, 1H), 7.34-7.35 (d, J = 5 Hz, 2 H). ^13^C NMR (125 MHz, CDCl_3_) δ 146.92, 129.64, 118.73, 115.45.


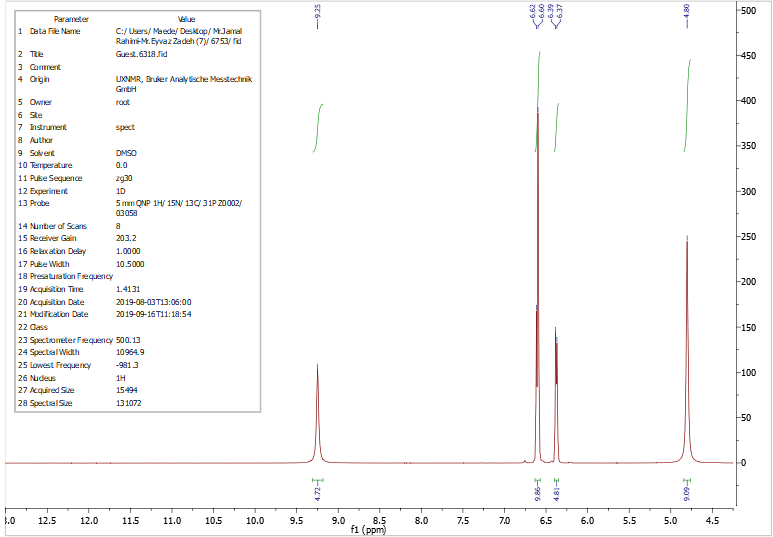


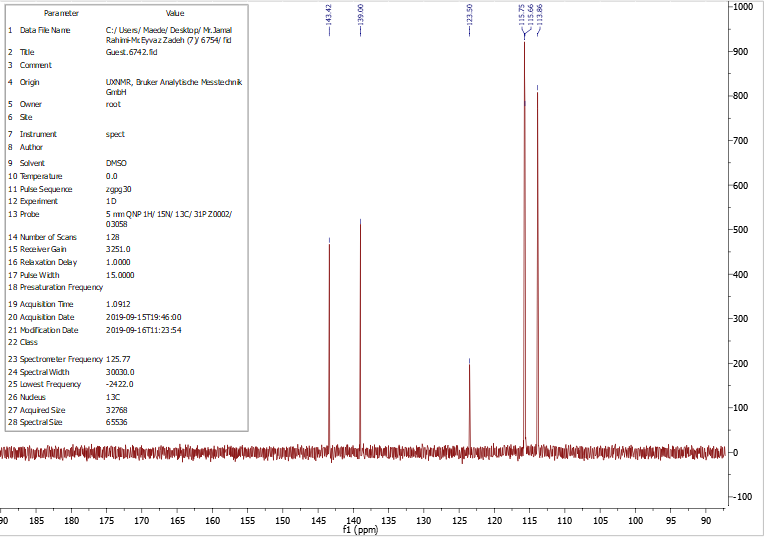


**Figure S2.** ^1^H-NMR and ^13^C-NMR spectra and spectral data of 2-amino-4-chloro-phenol.

Sepectral data: ^1^H NMR (500 MHz, DMSO) δ 9.25 (s, 1H), 6.61 (m, 1H), 6.61 (m, 1H), 6.38 (m, 1H), 4.80 (s, 2H). ^13^C NMR (125 MHz, DMSO) δ 143.42, 139.00, 123.50, 115.75, 115.66, 113.86.

**Figure S3.** ^1^H-NMR and ^13^C-NMR spectra and spectral data of benzene-1,3-diamine.

Spectral data: ^1^H NMR (500 MHz, CDCl_3_) δ 6.96-6.99 (m, 1 H), 6.15–6.13 (d, J = 7.8 Hz, 2 H), 6.03 (s, 1 H), 3.59 (s, 4 H). ^13^C NMR (500 MHz, CDCl_3_) δ 147.78, 130.34, 106.25, 102.18.


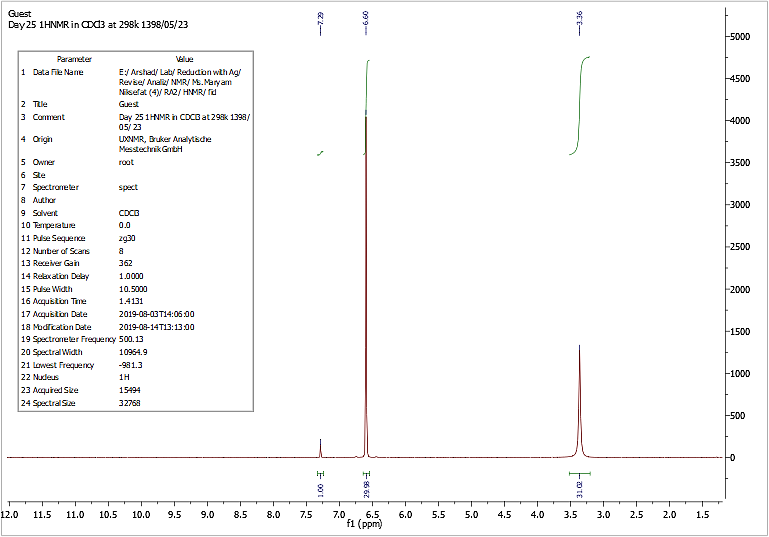


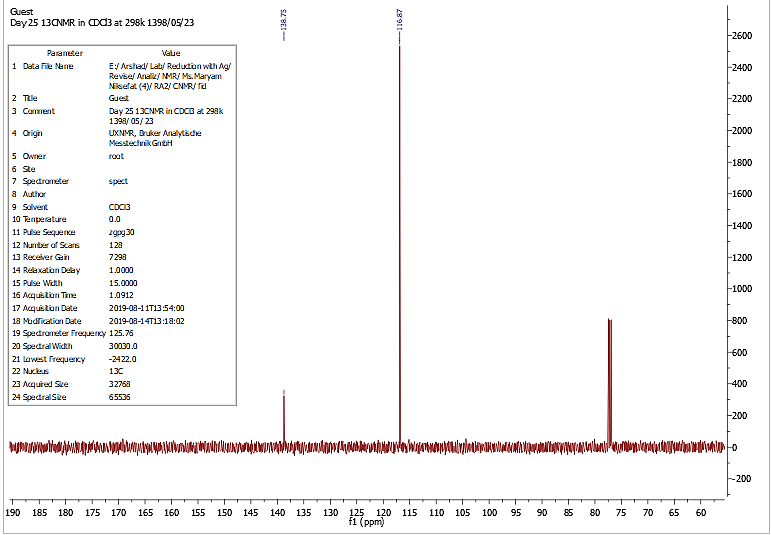


**Figure S4.** ^1^H-NMR and ^13^C-NMR spectra and spectral data of benzene-1,4-diamine.

Spectral data: ^1^H NMR (500 MHz, CDCl_3_) δ 6.59 (s, 4 H), 3.36 (s, 4 H). ^13^C NMR (125 MHz, CDCl_3_) δ 138.75, 116.87.


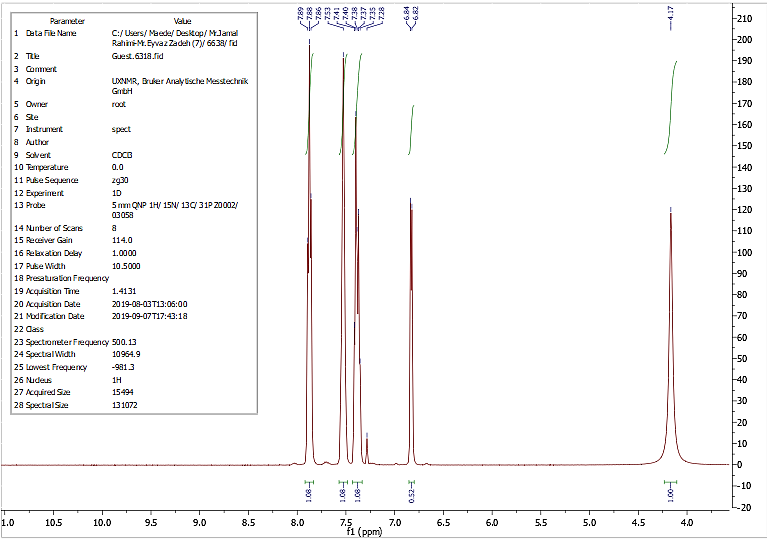


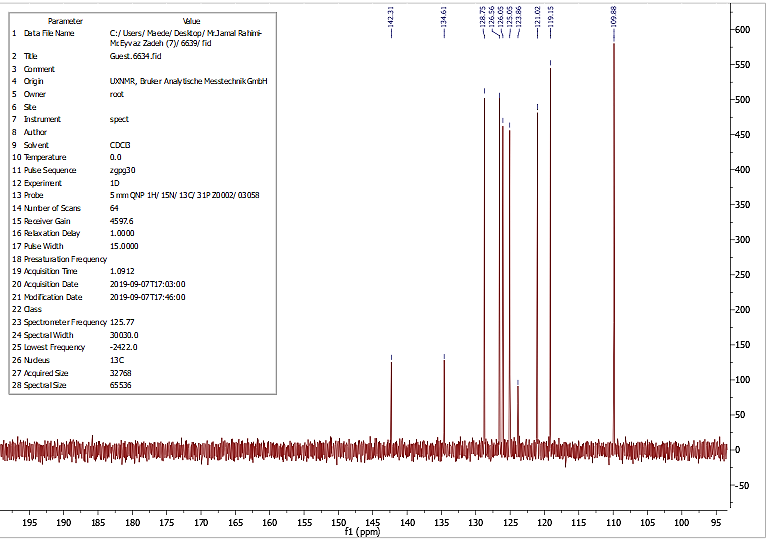


**Figure S5.** ^1^H-NMR and ^13^C-NMR spectra and spectral data of 2-Naphthylamine.

Spectral data: ^1^H-NMR (500 MHz, CDCl_3_): δ = 7.88 (m, 2H), 7.53 (m, 2H), 7.38 (m, 2H), 6.83 (m, 1H), 4.17 (s, 2H). ^13^C-NMR (125 MHz, CDCl_3_): δ = 142.31, 134.61, 128.75, 126.56, 126.05, 125.05, 123.86, 121.02, 119.15, 109.88.

**Figure S6.** ^1^H-NMR and ^13^C-NMR spectra and spectral data of p-toluidine.

Spectral data: ^1^H NMR (500 MHz, CDCl_3_) δ 2.28 (s, 3H), 3.56 (s, 2H), 6.64-6.66 (d, J = 10 Hz, 2H), 7.00-7.02 (d, J = 10 Hz, 2H). ^13^C NMR (500 MHz, CDCl_3_) δ 20.60, 115.43, 127.96, 129.92, 143.97.


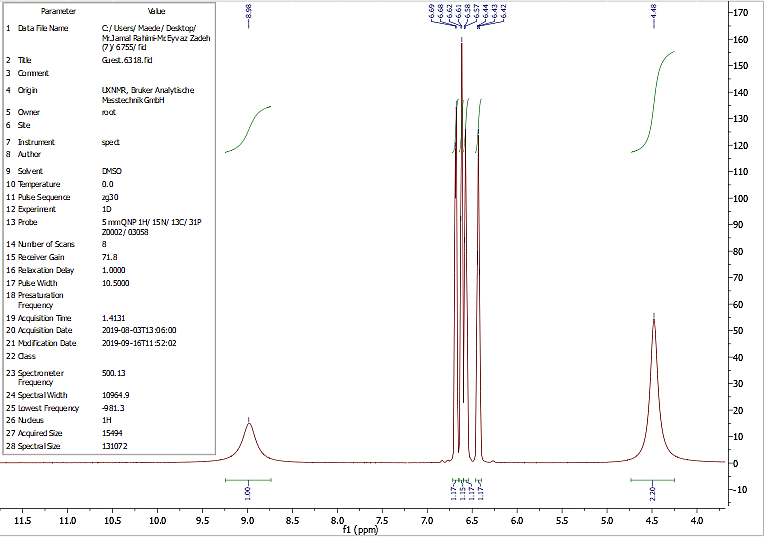


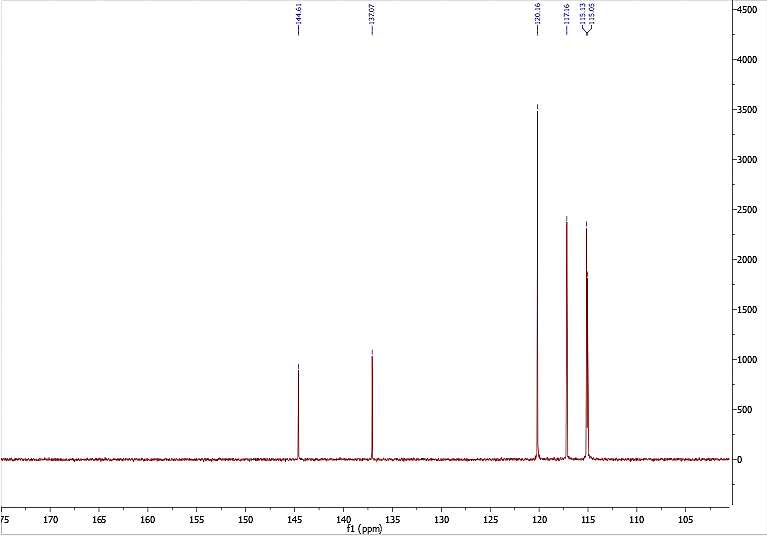


**Figure S7.** ^1^H-NMR and ^13^C-NMR spectra and spectral data of 2-aminophenol.

Spectral data: ^1^H NMR (500 MHz, DMSO) δ 8.98 (s, 1H), 6.68 (m, 1H), 6.61 (m, 1H), 6.57 (m, 1H), 6.43 (m, 1H), 4.48 (S, 2H). ^13^C NMR (125 MHz, DMSO) 144.51, 137.07, 120.16, 117.16, 115.13, 115.05.

**Figure S8.** ^1^H-NMR and ^13^C-NMR spectra and spectral data of 4-chloroaniline.

Spectral data: ^1^H NMR (500 MHz, CDCl_3_) δ 3.68 (s, 2H), 6.61-6.63 (d, J = 10 Hz, 2H), 7.12-7.14 (d, J = 10 Hz, 2H). ^13^C NMR (500 MHz, CDCl_3_) δ 116.42, 123.20, 129.28, 145.22.


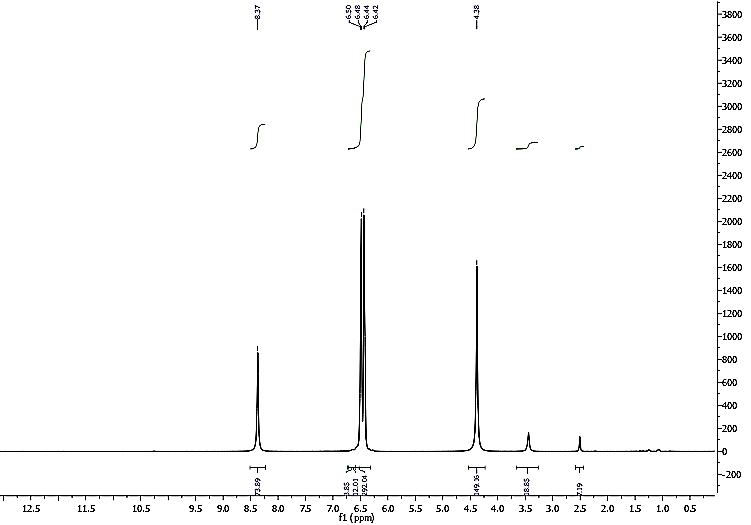


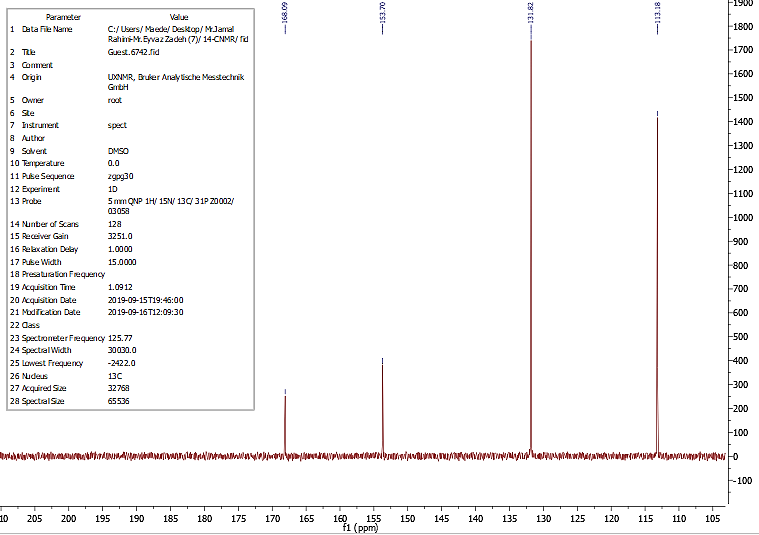


**Figure S9.** ^1^H-NMR and ^13^C-NMR spectra and spectral data of 4-amino-phenol.

Spectral data: 4-Aminophenol: white solid, ^1^H NMR (500 MHz, DMSO): δ (ppm) = 4.38 (2H, s, NH_2_), 6.42–6.44 (2H, d, J=10 Hz, H–Ar), 6.48–6.50 (2H, d, J=10 Hz, H–Ar), 8.37 (1H, s, OH). ^13^C NMR (125 MHz, DMSO) δ 168.09, 153.70, 131.82, 113.18.


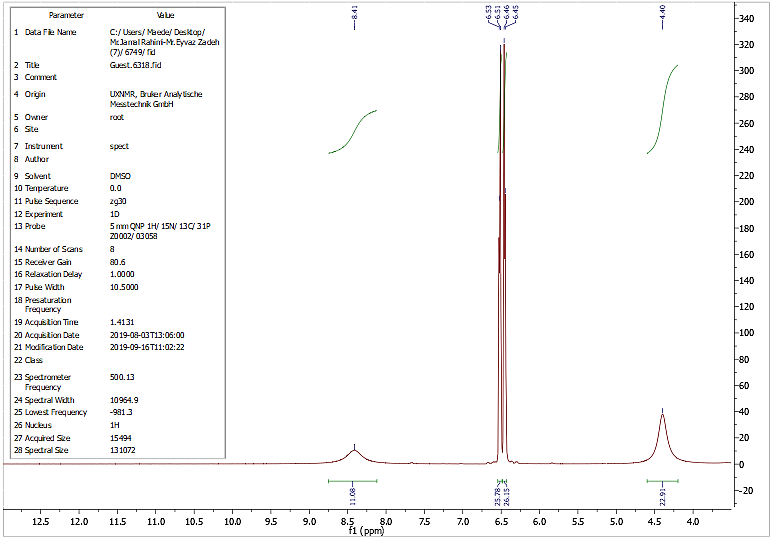


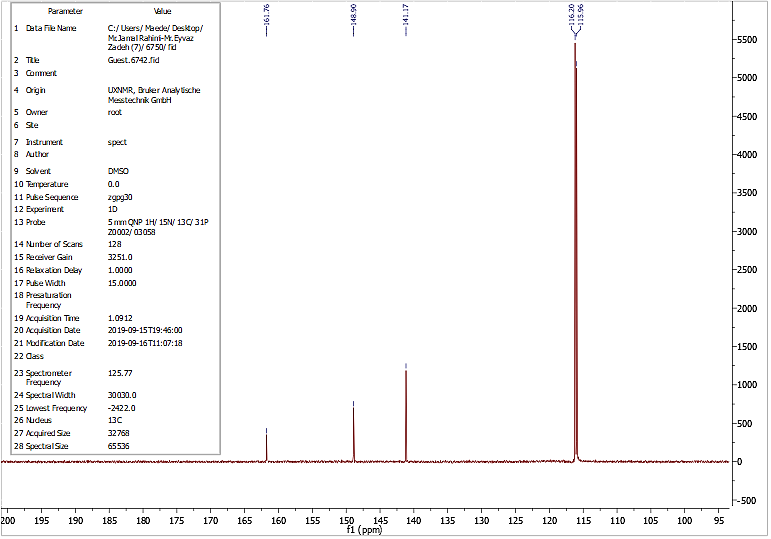


**Figure S10.** ^1^H-NMR and ^13^C-NMR spectra and spectral data of 4-amino-benzonic acid.

Spectral data: ^1^H NMR (500 MHz, DMSO) δ 8.41 (s, 1H), 6.53-6.51 (d, J = 10 Hz, 2H), 6.46-6.45 (d, J = 5 Hz, 2H), 4.40 (s, 2H). ^13^C NMR (125 MHz, DMSO) δ 161.76, 148.90, 141.17, 116.20, 115.96.

The histogram of the particle size distribution is provided. Figure (a) shows that most Fe_3_O_4_ magnetic nanoparticles were prepared in a 50-60 nm size range. The average size of the nanoparticles’ diameter was 60.49 nm. As demonstrated in Figure (b), the particle size distribution remains uniform and in the same size range. The average diameter of the particles was 62.41 nm.


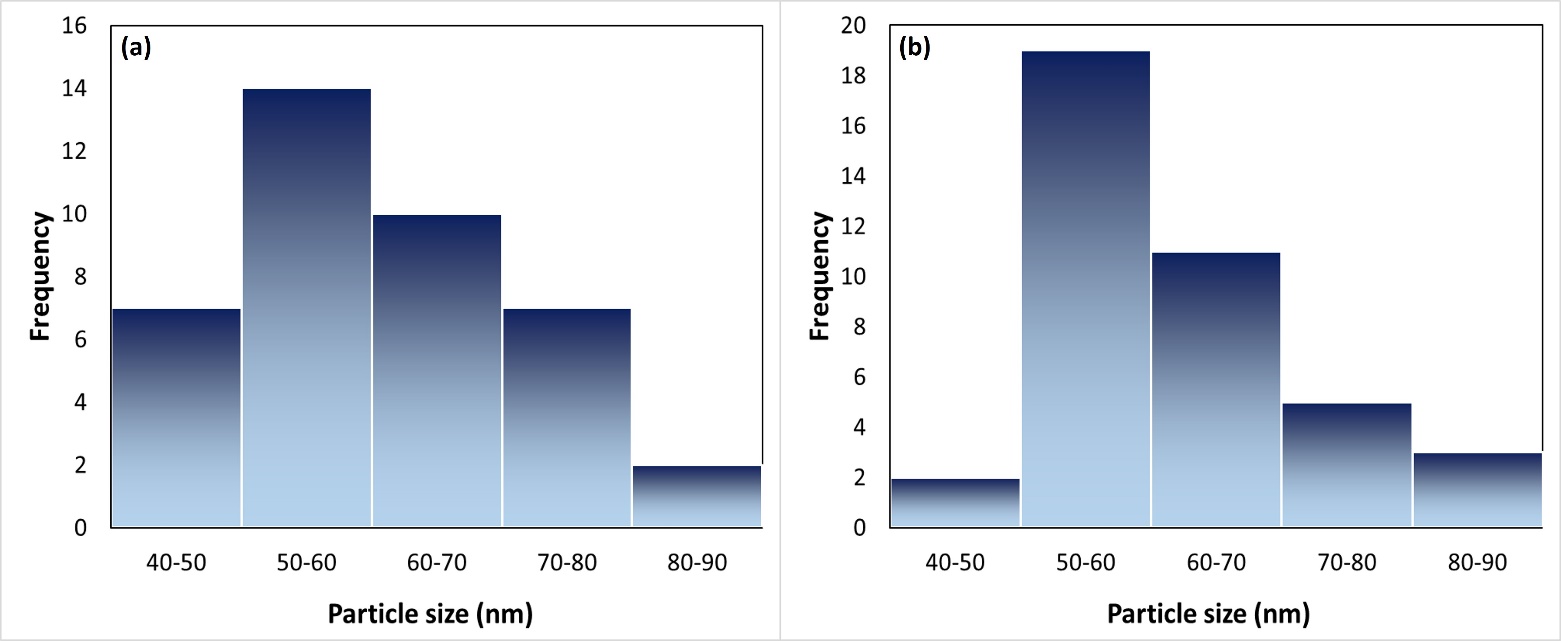


**Figure S11.** The Number-frequency histogram of (a) Fe_3_O_4_ magnetic nanoparticles and (b) Fe_3_O_4_ magnetic nanoparticles in Fe_3_O_4_@Cur/Mel-Ag magnetic nanocomposite.


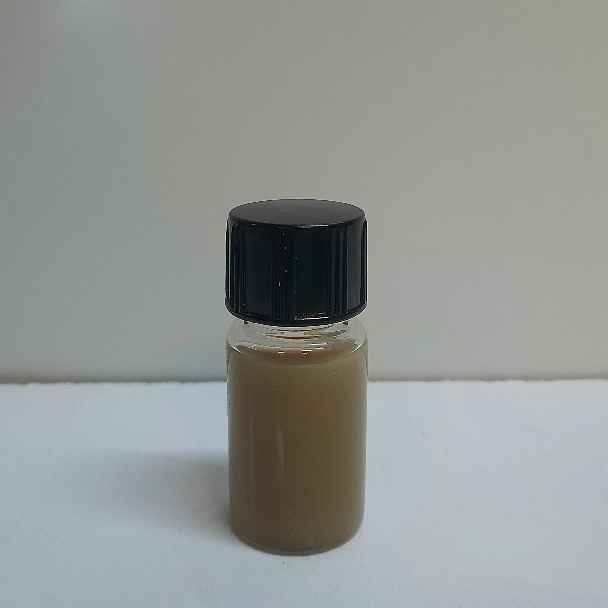


**Figure S12.** Filtered Fe_3_O_4_@Cur/Mel-Ag nanocatalyst solution after the NB reduction reaction.

**
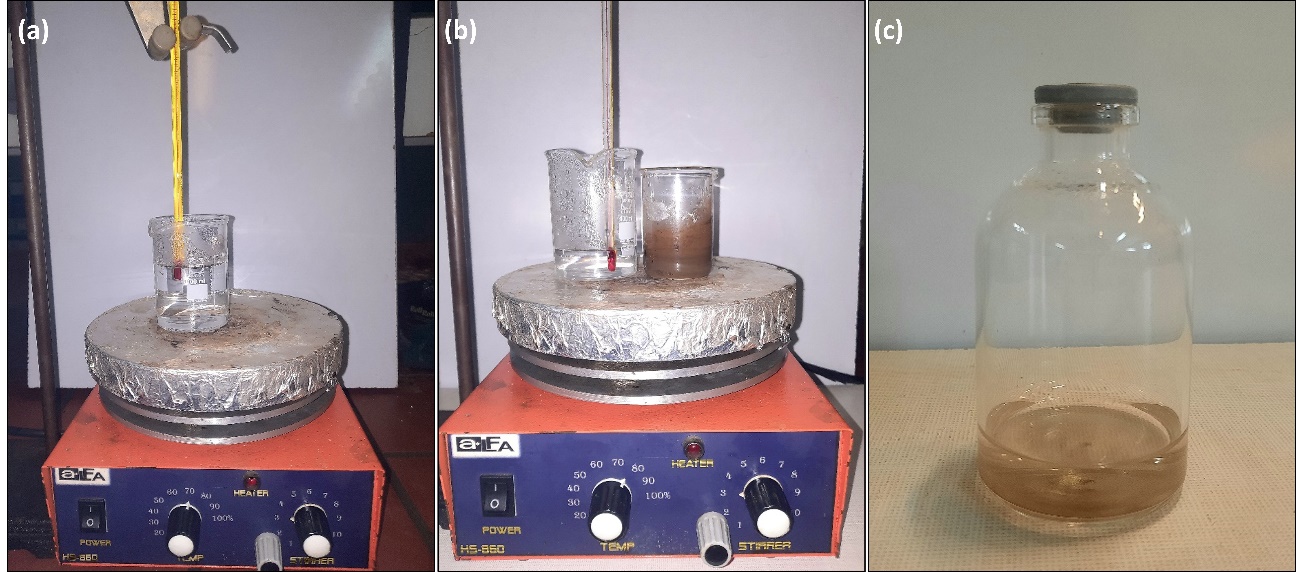
**

**Figure S13.** (a) The boiling solvent, (b) The catalyst containing solvent, (c) The filtered solution.
